# tRigon: an R package and Shiny App for integrative (path-)omics data analysis

**DOI:** 10.1186/s12859-024-05721-w

**Published:** 2024-03-05

**Authors:** David L. Hölscher, Michael Goedertier, Barbara M. Klinkhammer, Patrick Droste, Ivan G. Costa, Peter Boor, Roman D. Bülow

**Affiliations:** 1 Institute of Pathology, RWTH Aachen University Clinic, Aachen, Germany; 2 Institute for Computational Genomics, RWTH Aachen University Clinic, Aachen, Germany; 3 Department of Nephrology and Immunology, RWTH Aachen University Clinic, Aachen, Germany

**Keywords:** Data exploration, Statistics, User interface, Pathomics

## Abstract

**Background:**

Pathomics facilitates automated, reproducible and precise histopathology analysis and morphological phenotyping. Similar to molecular omics, pathomics datasets are high-dimensional, but also face large outlier variability and inherent data missingness, making quick and comprehensible data analysis challenging. To facilitate pathomics data analysis and interpretation as well as support a broad implementation we developed tRigon (Toolbox foR InteGrative (path-)Omics data aNalysis), a Shiny application for fast, comprehensive and reproducible pathomics analysis.

**Results:**

tRigon is available via the CRAN repository (https://cran.r-project.org/web/packages/tRigon) with its source code available on GitLab (https://git-ce.rwth-aachen.de/labooratory-ai/trigon). The tRigon package can be installed locally and its application can be executed from the R console via the command ‘tRigon::run_tRigon()’. Alternatively, the application is hosted online and can be accessed at https://labooratory.shinyapps.io/tRigon. We show fast computation of small, medium and large datasets in a low- and high-performance hardware setting, indicating broad applicability of tRigon.

**Conclusions:**

tRigon allows researchers without coding abilities to perform exploratory feature analyses of pathomics and non-pathomics datasets on their own using a variety of hardware.

**Supplementary Information:**

The online version contains supplementary material available at 10.1186/s12859-024-05721-w.

## Introduction

Histologic tissue analysis is vital for investigating disease states, understanding pathophysiological mechanisms and guiding diagnostics. Recent technological developments in digital and computational pathology enabled automated large-scale histopathology analyses [1–4]. The expansion of digital pathology has especially been fueled by deep learning-based workflows [5–8]. While end-to-end approaches focus on direct clinically or diagnostically actionable outputs, pathomics uses large-scale extraction of explainable, quantitative color or geometric features (e.g., the circularity) from histological structures identified using semantic segmentation for data mining of histopathology [9–14]. This approach is similar to molecular omics approaches and aims to better understand morphology by generating morphometric features for relevant tissue structures, allowing exploratory analyses [15]. The extracted features could be integrated into clinical decision-making, e.g., for patient risk stratification [16] or outcome prediction [17, 18]. Pathomics data can be generated with comparatively little cost in comparison to other omics methods, enabling broad implementation in many research groups. This makes pathomics analyses especially interesting for biomedical researchers performing histological analyses, but the datasets can be challenging for established conventional omics workflows due to large outlier variability and missingness caused by inconsistent occurrences of analyzed structures. In addition, biomedical researchers who mostly perform tissue-based analyses often lack the specific coding skills needed for analyzing pathomics data and streamlining time-intensive data curation processes [19]. For these reasons, we have developed an R shiny application — tRigon (Toolbox foR InteGrative (path-)Omics data aNalysis) — to make exploratory pathomics data analyses more open, accessible and feasible to researchers and clinicians. While tRigon was mainly designed for its application to pathomics data, it is also suitable for analysis of other high- or low-dimensional data such as molecular omics or medical datasets.

## Implementation

**Fig. 1:**
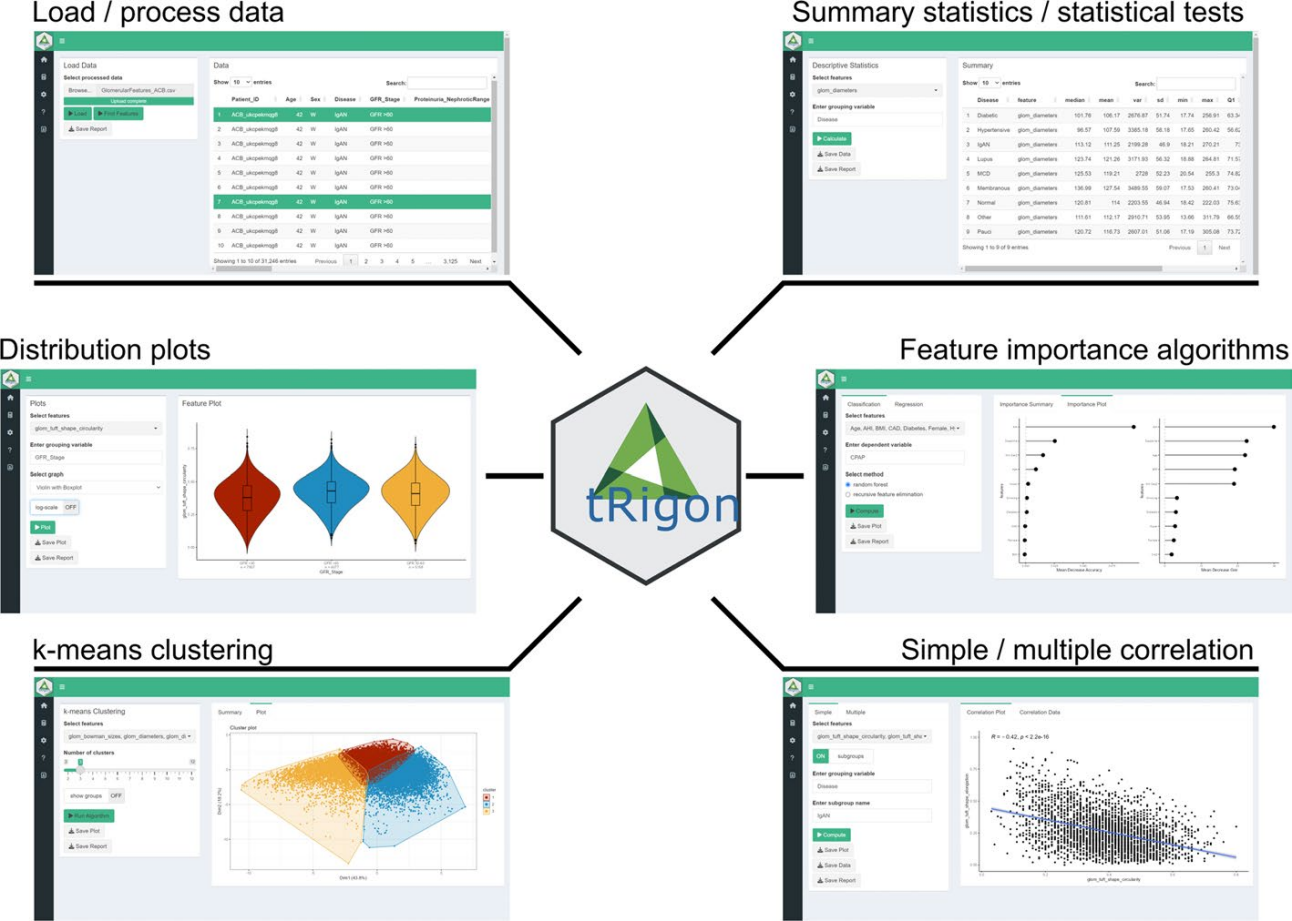
Overview of the available tRigon functions with their respective appearance in the user interface (ui)

tRigon is a Shiny application [20] built in the R framework [21] and is available both on CRAN (https://cran.r-project.org/web/packages/tRigon) and GitLab (https://git-ce.rwth-aachen.de/labooratory-ai/trigon). It includes various functions such as descriptive statistics, statistical tests and visualizations for analyzing large and complex datasets (Fig. 1). tRigon was tested on Windows, Linux and MacOS.

Pathomics datasets typically consist of multiple .csv files, for example generated by our previously published framework for large-scale histomorphometry (FLASH) [9]. The datasets include structural morphometric measurements (e.g., diameter, area or shape-descriptors) for major histological compartments and structures. For large human cohorts or animal experiments, this can be challenging to analyze. Furthermore, the data needs to be integrated with additional metadata. For human specimens, all tissue pieces on a slide typically belong to the same case and share the same clinical information (e.g., two biopsy cores) while some slides from animal experiments contain samples from multiple experimental conditions, e.g., multiple specimens from various animals or a diseased specimen and its internal or contralateral control tissue on the same slide. Additionally, pathomics data can be analyzed on the specimen level (e.g., a single human pathology case) or with single structure resolution.

tRigon can aggregate large amounts of pathomics files based on metadata with other (e.g., clinical) information of the analyzed samples. Based on the desired analysis the application allows for human- or animal-type data workflows and supports specimen or structure level calculations.

**Fig. 2:**
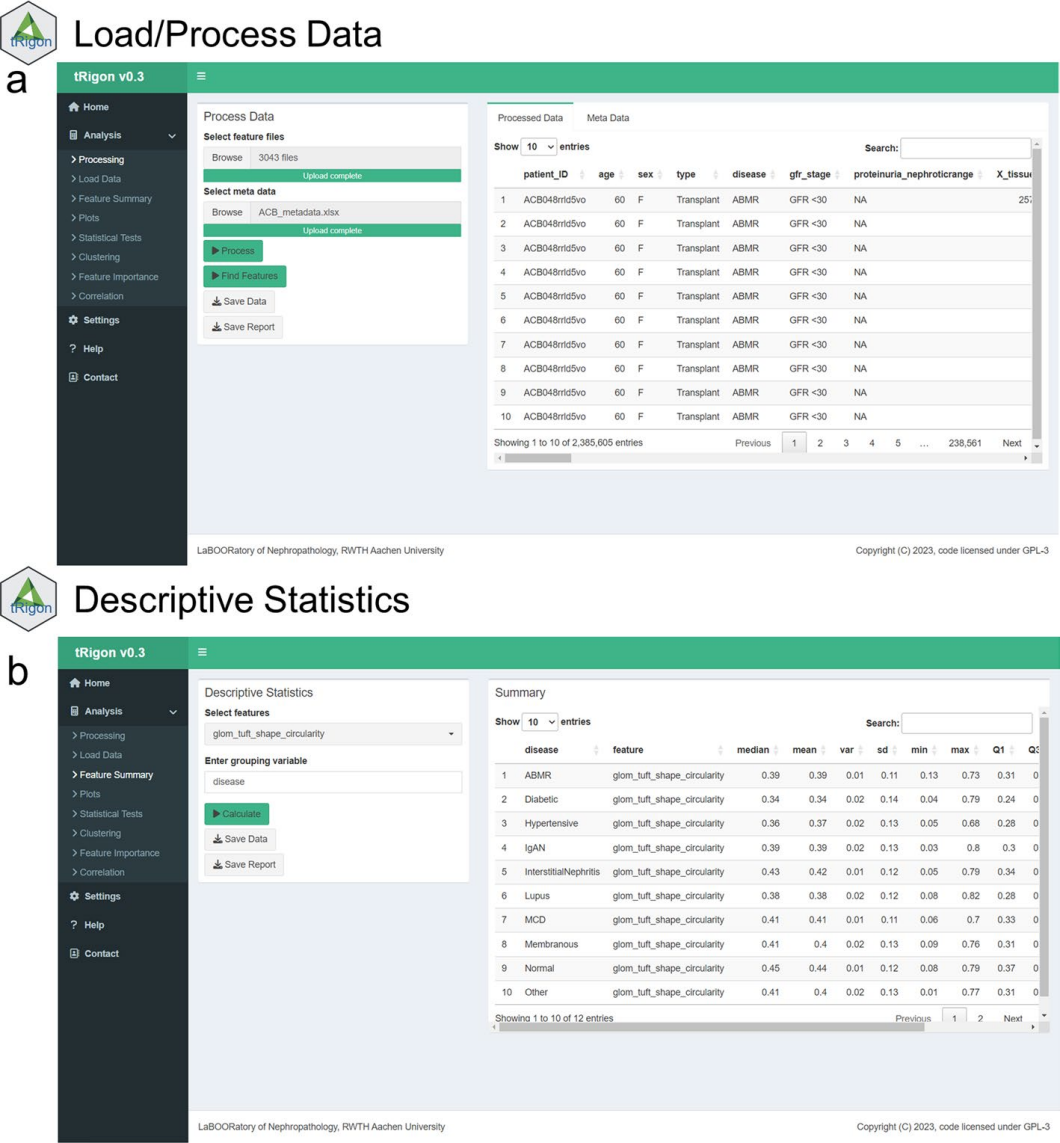
User interfaces of the **a** load/process data and **b** descriptive statistics tabs

**Fig. 3:**
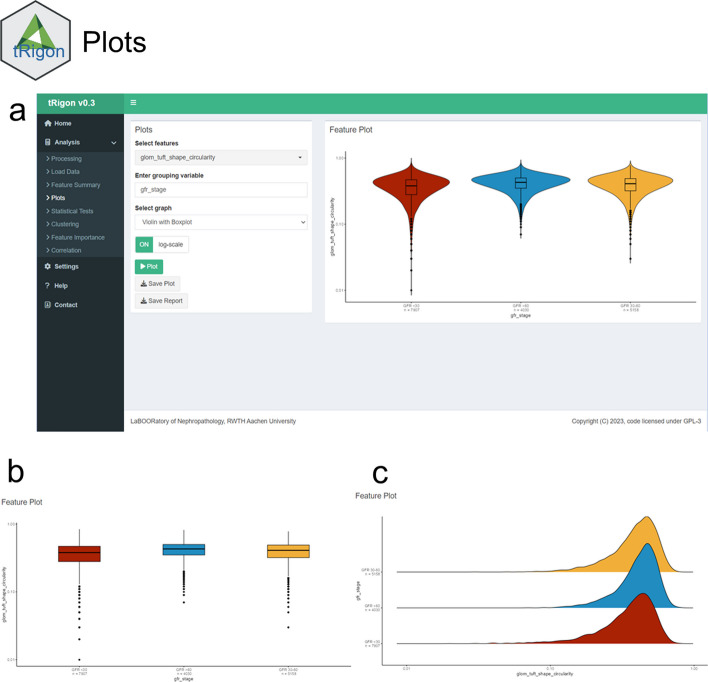
User interface of the **a** plotting tab. **b** example box plot and **c** example ridgeline plot with logarithmic scale set to “on”

**Fig. 4:**
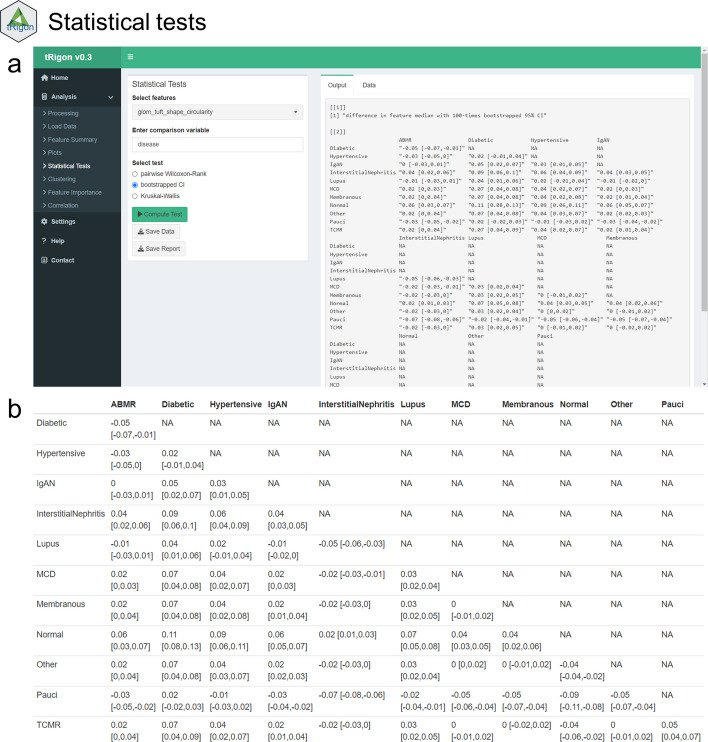
User interface of the **a** descriptive statistics tab and **b** example output for the 100-times bootstrapped comparisons of medians with 95% confidence intervals for the feature “glom_tuft_shape_circularity” stratified by histopathological diagnoses in the AC_B cohort. Additional selectable tests include pairwise Wilcoxon-rank test and Kruskal–Wallis test

**Fig. 5:**
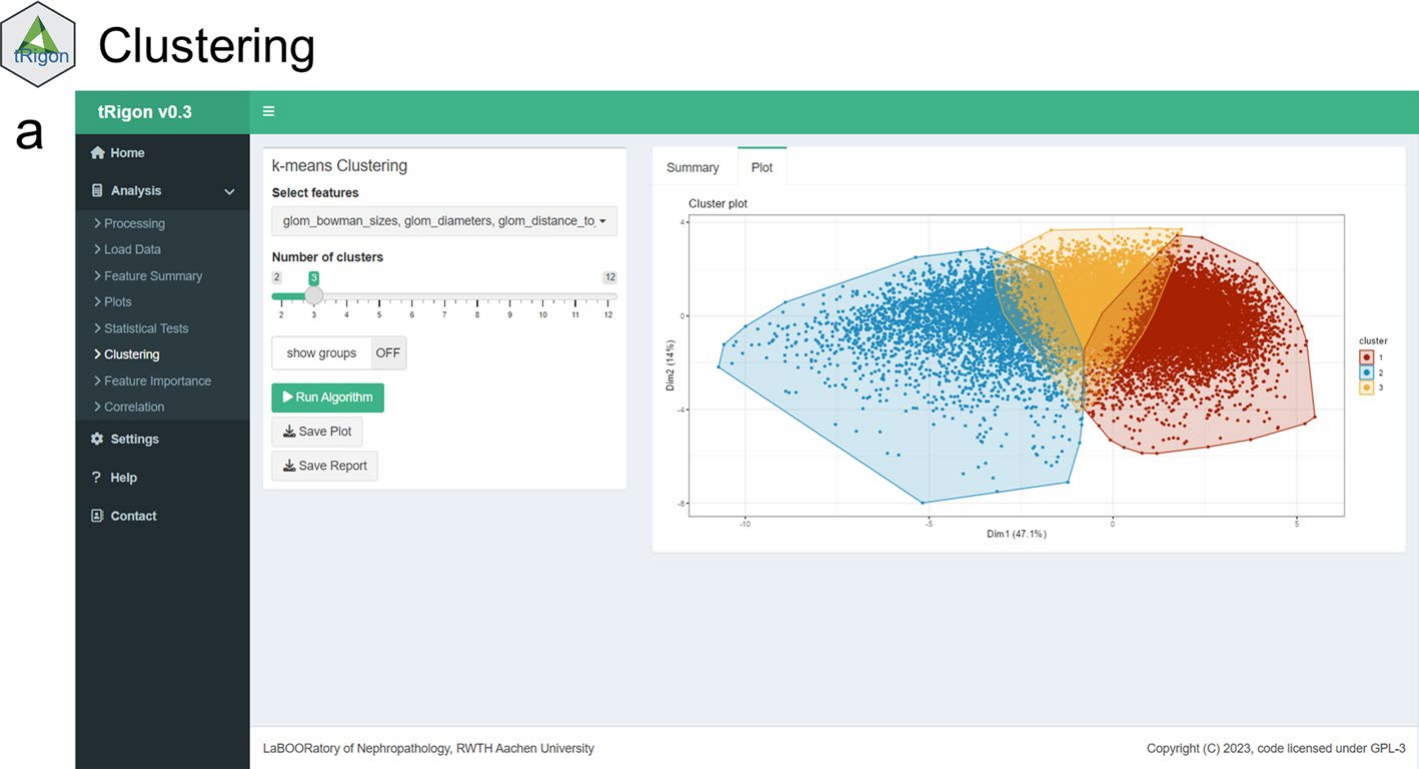
User interface of the **a** clustering tab. Features to be clustered can be selected, as well as the number of clusters and whether data points should be assigned to a group based on a grouping column in the metadata

**Fig. 6:**
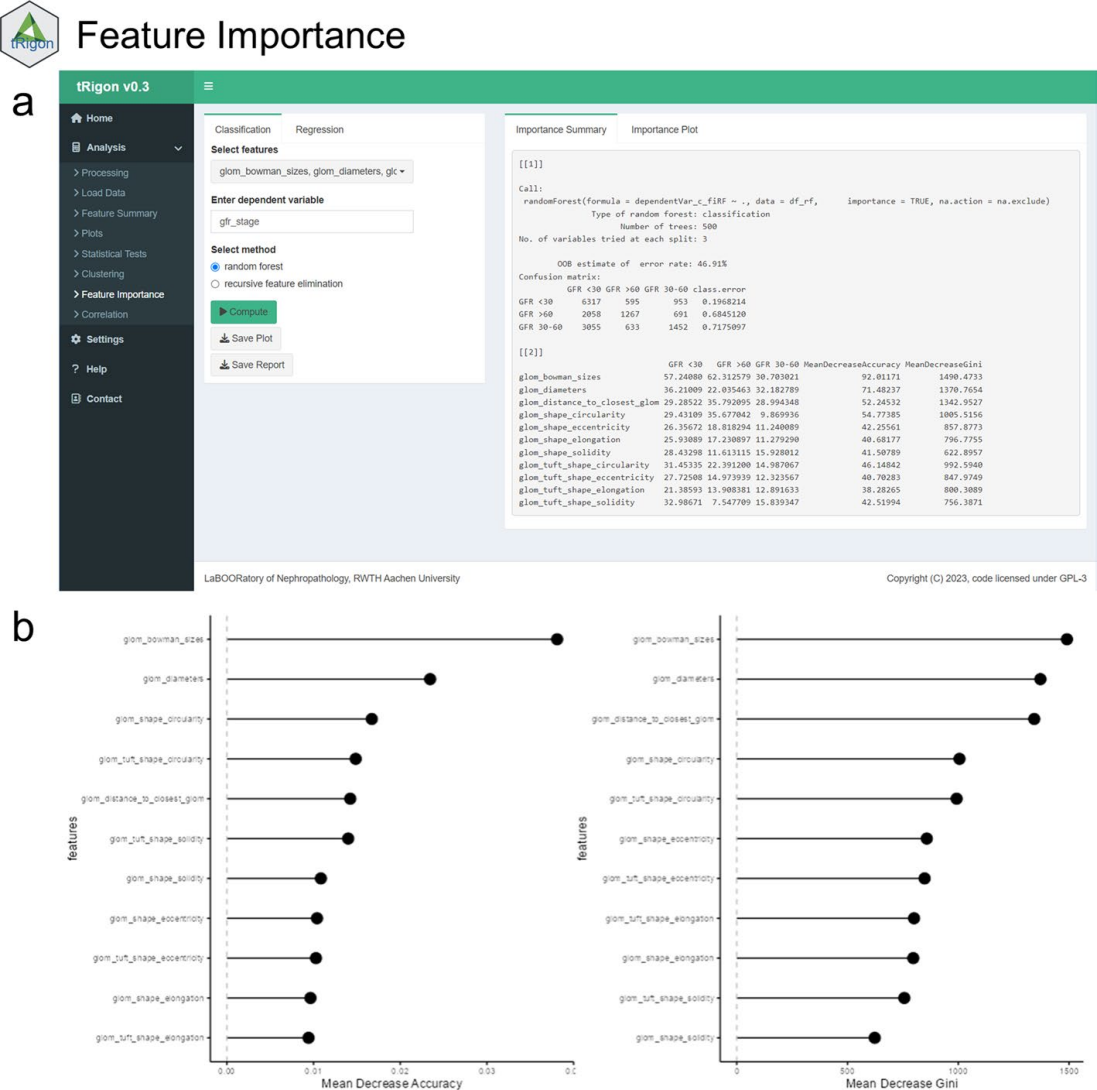
User interface of the **a** feature Importance tab. Features can be selected to perform random forest- or recursive feature-based importance analysis for classification and regression tasks. **b** Example feature importance plots showing mean decrease accuracy and mean decrease gini for the selected features and dependent variable

**Fig. 7:**
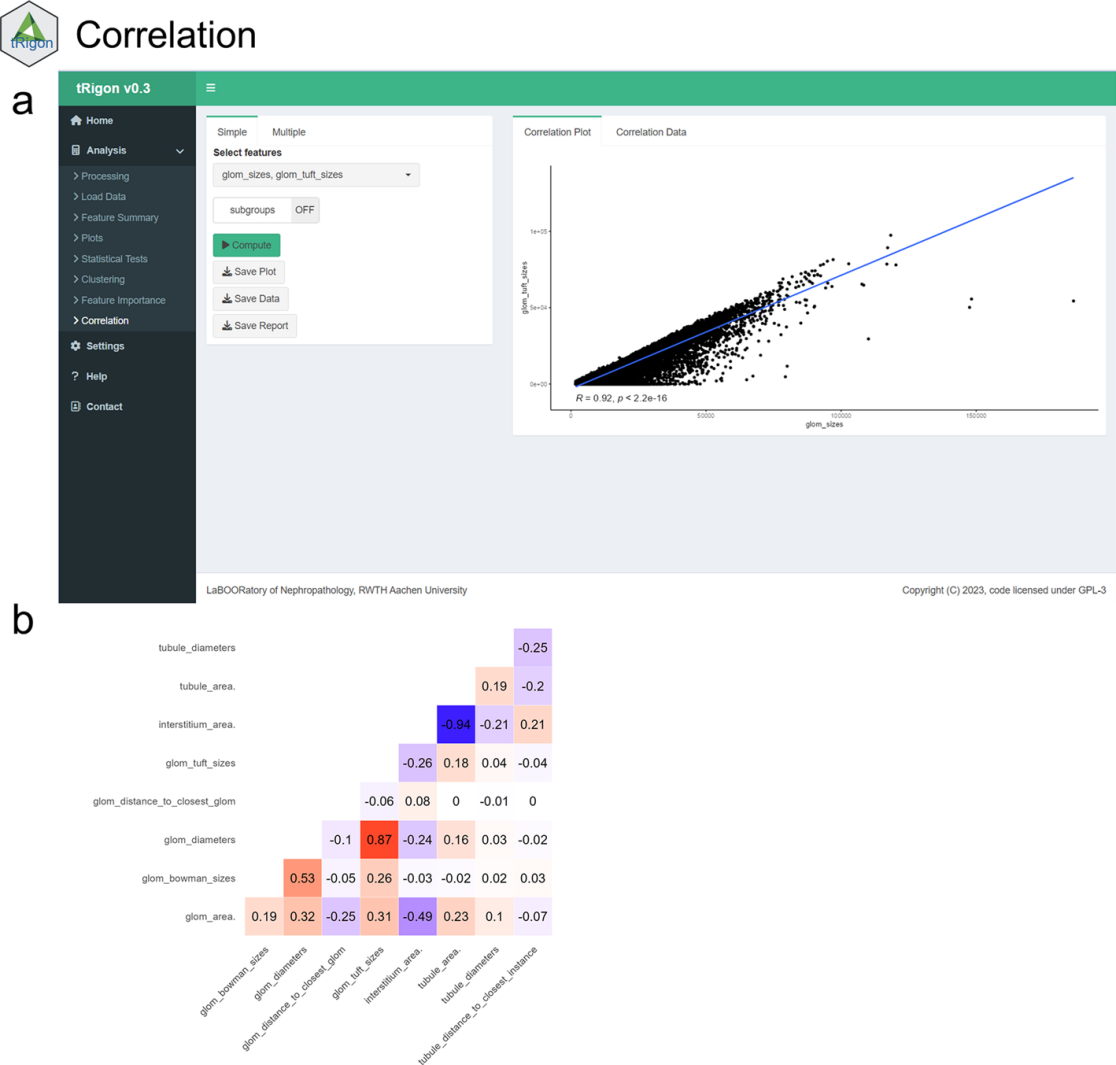
User interface of the **a** correlation tab. Features can be selected to perform single- or multiple correlation showing a single correlation plot as an example output. **b** Example multiple correlation visualized as a correlation matrix

For the aggregated feature files or own loaded datasets, tRigon provides users with a toolbox of different analytical methods, i.e., statistics, data visualizations and machine learning algorithms (Table 1). Each analysis tool represents a tab in the application and consists of an easily understandable user interface (Figs. 2, 3, 4, 5, 6, 7). tRigon users can tailor all functions to their specific needs by choosing from various statistical tests, distribution plots, machine learning methods and output style options. To effectively handle heterogeneous datasets, missingness is automatically reported in the application, non-normally distributed features are supported by multiple non-parametric tests and outliers can be scaled in plots accordingly. Additionally, the application includes a help section with instructions and common pitfalls. All processed data, generated plots and computed statistical tests can be downloaded if desired. To enable reproducible analyses across user sessions and to keep a record of results tRigon can generate and save markdown-based.html-reports including all relevant inputs (e.g., chosen features and group column, plot selection, etc.) and outputs for each task (Table 1). A full example analysis is provided in the supplementary material (Additional file 9: Table S1–S3 and Additional file 9: Figs. S1–S4).

**Table 1:** tRigon functions with explanations

Section	Function	Explanation
Data	Processing data	tRigon can process .csv files of pathomics data together with provided experiment/clinical data meta files. tRigon aggregates pathomics files and assigns them to the provided labels from the metadata. Users can choose between processing human or animal experiment data with calculations on specimen or single-structure level
	Loading data	tRigon can also be used to load other data (e.g., other omics datasets) or already processed pathomics files
Statistics	Descriptive statistics	Based on a provided group label tRigon can calculate summary statistics (e.g., mean, median, standard deviation, interquartile range) for each chosen feature
	Statistical tests	tRigon supports a range of simple non-parametric statistical tests such as: (1) pairwise Wilcoxon-rank tests with Bonferroni correction for multiple testing (2) Kruskal–Wallis tests (3) differences in median with bootstrapped confidence intervals for each desired feature and provided group label
	Correlation	Simple and multiple correlations based on the Pearson-correlation coefficient can be calculated and visualized as a scatter plot or correlation matrix for each chosen feature. Users can also specify a group and subgroup for specific correlation analysis
	Machine learning	For calculation of feature importance tRigon supports random forests and recursive feature elimination (RFE) for classification and regression of chosen features based on a selected dependent variable. For RFE users can also specify the number of folds for cross-validation as well as repeats
Plots & Visualisation	Distribution plots	Based on a provided group label tRigon plots selected feature distributions in a variety of plots: (1) violin plots, with/without box plots (2) box plots (3) ridgeline plots
	Clustering	tRigon supports k-means clustering for selected variables. Groups can also be plotted within a separate legend
Logs	Markdown reports	For each function tRigon users can download a markdown report in.html format including all relevant inputs and outputs of the application

In addition to running tRigon locally via the R console, the application is freely available online in the ShinyApps.io workspace (https://labooratory.shinyapps.io/tRigon), albeit the memory size for free use is limited to 1 GB of Random-Access Memory (RAM). Therefore, users are advised to process and analyze computationally expensive files such as large pathomics datasets locally.

## Results

Nine datasets from different platforms were acquired to demonstrate the effectiveness, versatility, and limitations of tRigon. Five of those are pathomics datasets including four human kidney cohorts and one animal experiment for 2,8-dihydroxyadenine crystal nephropathy, a mouse model for diet-induced tubulointerstitial fibrosis and scarring [22]. The human kidney datasets consist of two in-house biopsy (AC_B) and nephrectomy (AC_N) datasets [9] as well as the freely available *Kidney Precision Medicine Project* (KPMP) [23] and *Human BioMolecular Atlas Program* (HuBMAP) [24] datasets containing kidney biopsies and nephrectomies. Furthermore, we analyzed freely available aggregated specimen level pathomics data from a recent study on breast cancer, replicating their results (Additional file 9: Table S4 and Additional file 9: Figs. S5–S9) [14]. In total, the four human pathomics datasets include 3,287 instance level files with a total file size of 312.7 MB while the 2,8-dihydroxyadenine crystal nephropathy pathomics dataset consists of 9 files with a total file size of 13.0 MB. The aggregated breast cancer histomics data file contains a file size of 7.55 MB. Furthermore, three freely available non-pathomics medical datasets [25–27] with a total file size of 4.62 MB from the *Teaching of Statistics in the Health Sciences* (TSHS) Resources Portal were included.

Computation time was evaluated using two different settings, representing a high- and low-resource setting and three datasets with different sizes (Table 2). Setting A refers to running the application on a hybrid tablet-notebook (Intel Pentium CPU 1.60 GHz with 8 GB RAM) while setting B refers to running tRigon on a workstation (Intel Xeon Gold 6128 CPU 3.40 GHz, 128 GB RAM). In general, running tRigon on a workstation was faster, but computation times were still quick, and performance was smooth when running the app on setting A, even for large datasets (Table 2). Regardless of hardware tRigon was especially fast for statistical analysis (summary statistics, pairwise Wilcoxon-rank tests, and correlations) and visualizations (distribution plots, scatter plots, and correlation matrices). Processing data frames and machine learning algorithms remained more time-consuming operations, as expected (Table 2).

**Table 2:** tRigon runtime based on data frame size and computational setting

Task	Setting A	Setting B
Processing data		
Small	–	–
Medium	39,280 ms	20,190 ms
Large	283,870 ms	114,790 ms
Loading data		
Small	1520 ms	1290 ms
Medium	5370 ms	4460 ms
Large	47,550 ms	29,740 ms
Summary statistics (1 feature)		
Small	650 ms	230 ms
Medium	1320 ms	1200 ms
Large	13,590 ms	4020 ms
Distribution plots (1 feature, violin plot, no log-scale)		
Small	2410 ms	1290 ms
Medium	1980 ms	1220 ms
Large	5780 ms	3280 ms
Statistical tests (1 feature, pairwise-Wilcoxon test)		
Small	170 ms	70 ms
Medium	290 ms	80 ms
Large	1180 ms	570 ms
k-means clustering (6 features, 4 cluster, no groups)		
Small	1450 ms	840 ms
Medium	2970 ms	730 ms
Large	5590 ms	1930 ms
Feature importance (classification, 6 features, random forest)		
Small	2000 ms	1040 ms
Medium	6090 ms	2990 ms
Large	43,980 ms	22,870 ms
Correlation matrix (multiple corr., 6 features, no subgroups)		
Small	1220 ms	510 ms
Medium	1460 ms	460 ms
Large	1570 ms	710 ms

All tasks were monitored with three dataframes (small: 281 rows, 36 columns, 55.9 KB size; medium: 211,287 rows, 53 columns, 47.7 MB size; large: 2,385,605 rows, 42 columns, 228 MB size) and in two computational settings (A: Intel Pentium CPU 1.60 GHz, 8 GB RAM; B: Intel Xeon Gold 6128 CPU 3.40 GHz, 128 GB RAM). The small dataframe is a medical dataset (which cannot be processed) while the medium and large dataframes are pathomics datasets

*ms* milliseconds, *log* logarithmic, *corr.* correlation

## Discussion

tRigon is a user-friendly Shiny application for high-throughput, simple and reproducible analysis of high-dimensional data including pathomics datasets.

An obvious limitation of tRigon is that it is not designed to generate pathomics data. This means it cannot be used to directly investigate whole slide images and users must use another software. However, there are tools available that allow researchers, in some instances even without coding experience, to perform such analysis [28–31]. Another limitation is that tRigon is not designed as a full-scale statistical program, i.e., in-depth statistical analyses need to be performed with dedicated tools. However, the app allows adding new functionalities, potentially increasing the analytical tools in the future.

## Conclusion

With tRigon, users can easily and effectively summarize or correlate features, visualize distributions, statistically test hypotheses, implement machine learning algorithms and cluster data. Markdown reports can help users with documenting each analysis step. tRigon can further accelerate pathomics research and facilitate creating valuable readouts for large (path-)omics datasets. We will continuously update and expand tRigon in the future.

### Supplementary Information

**Additional file 1**. tRigon session report in html-format for a k-means clustering analysis including all inputs, setting options and outputs.

**Additional file 2**. tRigon session report in html-format for a correlation analysis including all inputs, setting options and outputs.

**Additional file 3**. tRigon session report in html-format for loading data into the application including a detailed description of the loaded data frame.

**Additional file 4**. tRigon session report in html-format for processing omics datasets including a detailed description of input files, processing settings and the processed data frame.

**Additional file 5**. tRigon session report in html-format for descriptive statistics including all inputs, setting options and outputs.

**Additional file 6**. tRigon session report in html-format for a feature importance analysis including all inputs, setting options and outputs.

**Additional file 7**. tRigon session report in html-format for feature plots including all inputs, setting options and outputs.

**Additional file 8**. tRigon session report in html-format for statistical testing including all inputs, setting options and outputs.

**Additional file 9**. Supplementary Material containing Supplementary Tables S1-S4 and Supplementary Figures S1-S9.

## Data Availability

Project name: tRigon; Project home page: https://git-ce.rwth-aachen.de/labooratory-ai/trigon; https://cran.r-project.org/web/packages/tRigon/index.html; https://labooratory.shinyapps.io/tRigon/; Operating system(s): Tested on Windows 10 & 11, Linux and MacOS; Programming language: R and CSS; Other requirements: all required packages will be installed when installing via the command “install.packages(“tRigon”); License: GNU GPLv3; Any restrictions to use by non-academics: as detailed in GNU GPLv3. All datasets included in this study are deposited in the tRigon repository (https://git-ce.rwth-aachen.de/labooratory-ai/trigon/-/blob/main/demo_data.zip) or the respective study repository and are freely available for users to test out the application.
