# Supplementary material for: tRigon: an R package and Shiny App for integrative (path-)omics data analysis
Source: BMC Bioinformatics. 2024 Mar 5;25:98. doi: 10.1186/s12859-024-05721-w (PMC10916305; doi:10.1186/s12859-024-05721-w)
Supplement: Supplementary file 1 — Additional file 1. tRigon session report in html-format for a k-means clustering analysis including all inputs, setting options and outputs. [file 12859_2024_5721_MOESM1_ESM.html]

Session Report - k-means Clustering


# Session Report - k-means Clustering


---

```
##  setting  value
##  version  R version 4.2.2 (2022-10-31 ucrt)
##  os       Windows 10 x64 (build 19045)
##  system   x86_64, mingw32
##  ui       RStudio
##  language (EN)
##  collate  German_Germany.1252
##  ctype    German_Germany.1252
##  tz       Europe/Berlin
##  date     2023-10-20
##  rstudio  1.4.1106 Tiger Daylily (desktop)
##  pandoc   2.11.4 @ C:/Program Files/RStudio/bin/pandoc/ (via rmarkdown)
```

features:

```
##  [1] "glom_bowman_sizes"             "glom_diameters"                "glom_distance_to_closest_glom"
##  [4] "glom_shape_circularity"        "glom_shape_eccentricity"       "glom_shape_elongation"        
##  [7] "glom_shape_solidity"           "glom_sizes"                    "glom_tuft_shape_circularity"  
## [10] "glom_tuft_shape_eccentricity"  "glom_tuft_shape_elongation"    "glom_tuft_shape_solidity"
```

compare groups enabled?

```
## [1] "Compare groups disabled."
```

data missingness:

```
## [1] "Warning: input vectors of unequal length - only complete rows can be analysed for k-means clustering. 813 rows with missing data excluded."
```

cluster output:

```
## K-means clustering with 5 clusters of sizes 205, 832, 443, 189, 839
## 
## Cluster means:
##   glom_bowman_sizes glom_diameters glom_distance_to_closest_glom glom_shape_circularity glom_shape_eccentricity
## 1        -0.6025273    -1.47701075                     0.5639573             -1.8680896              1.56943848
## 2         0.1093958     0.70875847                    -0.1216235              0.5708454             -0.92068285
## 3        -0.4864079    -0.99228415                     0.1056948             -0.5813843              0.72369130
## 4         2.1683850     0.87147647                     0.1280119             -0.8807709              0.21778152
## 5        -0.1929026    -0.01433495                    -0.1018326              0.3957496              0.09835196
##   glom_shape_elongation glom_shape_solidity glom_sizes glom_tuft_shape_circularity glom_tuft_shape_eccentricity
## 1             1.9660031          -2.2505456 -0.9569414                  -1.1427974                    1.4516053
## 2            -0.8147790           0.5086959  0.4729973                   0.4201451                   -0.9611207
## 3             0.6648891          -0.4085850 -0.8103152                  -0.3561069                    0.7842546
## 4             0.1674191          -0.3886184  1.4727197                  -1.1948601                    0.5537528
## 5            -0.0611711           0.3487234 -0.1391360                   0.3197814                    0.0595820
##   glom_tuft_shape_elongation glom_tuft_shape_solidity
## 1                  1.8023292               -1.5840672
## 2                 -0.8436594                0.5223438
## 3                  0.7473834               -0.4279033
## 4                  0.5099988               -1.0379662
## 5                 -0.1132699                0.3288206
## 
## Clustering vector:
##    [1] 5 2 2 5 3 3 3 2 2 5 5 5 3 3 5 3 5 2 2 5 3 3 3 5 2 2 2 5 3 5 5 3 2 2 2 3 3 5 3 1 2 5 2 2 5 5 2 2 5 2 3 5 3 2
##   [55] 2 2 5 3 5 5 1 2 5 2 2 2 5 3 2 2 5 2 3 5 3 5 2 5 5 2 5 3 2 2 2 5 5 2 2 3 5 5 2 2 5 2 4 2 2 2 2 2 2 2 2 2 2 2
##  [109] 2 2 5 5 2 5 3 2 5 3 2 2 2 5 5 4 5 2 5 3 5 2 2 2 2 3 2 5 5 2 2 4 5 5 5 5 2 2 5 2 3 2 2 2 2 5 5 5 2 2 5 5 2 2
##  [163] 2 2 5 5 2 5 3 5 5 5 3 2 5 2 2 2 5 2 2 2 5 3 5 3 2 3 5 5 5 2 5 2 5 3 5 5 5 2 2 5 5 3 2 4 3 2 5 5 5 2 2 5 5 5
##  [217] 5 3 5 2 4 3 2 2 5 2 5 3 2 2 1 5 1 2 3 2 2 5 2 5 2 5 4 2 5 5 2 2 3 2 3 2 5 3 5 3 5 5 2 2 2 2 2 2 5 5 2 5 2 2
##  [271] 5 3 5 5 2 4 2 2 5 5 5 2 2 3 2 2 5 2 2 2 5 3 2 5 2 5 5 2 3 5 4 5 2 5 2 5 2 2 2 5 2 5 2 1 2 4 2 3 1 2 2 5 5 2
##  [325] 1 4 3 2 5 2 5 5 5 5 2 5 2 3 5 5 2 5 4 3 2 2 2 2 2 2 2 5 5 2 4 2 2 2 3 5 2 5 2 5 5 2 2 2 2 5 2 2 2 2 2 5 5 2
##  [379] 2 5 2 3 3 1 2 5 2 5 2 5 5 5 2 2 3 3 3 5 5 2 3 2 2 2 5 2 2 3 2 2 3 3 3 5 5 3 2 5 5 2 5 4 5 2 5 5 5 5 5 5 3 2
##  [433] 2 2 5 2 2 3 2 5 2 2 5 5 3 3 5 2 5 2 2 2 3 2 5 5 5 2 5 2 5 5 5 5 3 3 2 3 3 5 5 5 5 5 5 2 2 3 5 2 2 2 5 5 2 5
##  [487] 5 2 2 2 5 5 2 5 5 5 3 5 2 5 3 2 4 5 2 3 2 5 5 5 5 4 5 2 5 5 5 5 5 2 5 3 2 3 3 1 2 5 5 5 5 2 2 5 5 5 3 5 2 1
##  [541] 5 3 5 5 2 3 3 3 5 5 2 5 2 5 3 5 2 5 5 5 5 2 5 5 5 5 2 3 5 4 5 5 5 2 4 5 3 2 2 5 2 5 5 5 3 5 2 5 2 2 2 3 2 5
##  [595] 5 5 5 2 2 5 2 2 2 3 1 5 5 5 5 2 5 2 5 5 2 5 2 2 5 5 5 2 5 4 5 5 3 2 5 3 5 2 5 5 3 5 2 3 5 2 5 5 2 2 5 5 3 5
##  [649] 5 3 2 3 5 2 4 2 5 5 2 5 5 2 2 2 2 5 2 5 3 2 5 3 5 2 2 3 5 1 3 5 3 3 2 5 2 3 2 2 3 2 2 5 5 2 2 2 5 5 3 3 3 5
##  [703] 5 3 2 5 2 5 2 5 5 5 5 2 5 5 5 5 2 3 3 5 5 2 5 5 2 2 5 1 5 2 2 2 5 2 2 2 2 2 5 2 3 5 5 3 3 2 2 5 2 5 5 3 5 5
##  [757] 5 5 2 3 2 5 2 5 5 5 2 3 5 3 1 5 3 5 2 2 1 5 2 5 3 2 5 2 3 2 1 3 3 3 5 5 3 3 2 5 2 2 2 3 5 2 2 5 2 2 5 5 2 2
##  [811] 2 5 5 2 2 2 3 5 5 3 5 2 5 5 5 5 2 5 5 5 5 2 2 2 5 3 2 5 5 5 5 5 5 2 5 4 5 5 3 2 5 5 2 5 2 5 2 5 2 5 5 4 5 2
##  [865] 5 2 4 3 2 5 5 1 5 3 5 5 1 5 5 3 5 5 5 1 5 2 5 2 2 5 1 4 5 2 1 2 5 2 3 5 5 2 2 2 4 2 5 4 4 5 2 2 1 5 2 2 2 5
##  [919] 2 2 2 5 5 5 5 2 5 5 2 3 3 5 5 5 5 2 5 1 3 5 3 3 2 2 5 3 4 5 5 4 3 4 2 5 2 2 5 5 2 3 2 1 4 5 2 1 2 4 1 4 3 1
##  [973] 2 4 5 1 5 2 2 3 4 3 3 1 1 1 4 3 5 3 5 4 2 1 4 5 5 3 5 4
##  [ reached getOption("max.print") -- omitted 1508 entries ]
## 
## Within cluster sum of squares by cluster:
## [1] 3335.537 3369.179 3086.491 2583.669 3046.473
##  (between_SS / total_SS =  48.7 %)
## 
## Available components:
## 
## [1] "cluster"      "centers"      "totss"        "withinss"     "tot.withinss" "betweenss"    "size"        
## [8] "iter"         "ifault"
```

cluster plot:
