# Supplementary material for: tRigon: an R package and Shiny App for integrative (path-)omics data analysis
Source: BMC Bioinformatics. 2024 Mar 5;25:98. doi: 10.1186/s12859-024-05721-w (PMC10916305; doi:10.1186/s12859-024-05721-w)
Supplement: Supplementary file 2 — Additional file 2. tRigon session report in html-format for a correlation analysis including all inputs, setting options and outputs. [file 12859_2024_5721_MOESM2_ESM.html]

Session Report - Correlation


# Session Report - Correlation


---

```
##  setting  value
##  version  R version 4.2.2 (2022-10-31 ucrt)
##  os       Windows 10 x64 (build 19045)
##  system   x86_64, mingw32
##  ui       RStudio
##  language (EN)
##  collate  German_Germany.1252
##  ctype    German_Germany.1252
##  tz       Europe/Berlin
##  date     2023-10-20
##  rstudio  1.4.1106 Tiger Daylily (desktop)
##  pandoc   2.11.4 @ C:/Program Files/RStudio/bin/pandoc/ (via rmarkdown)
```

```
## [1] "Multiple correlation with 9 features."
```

features:

```
## [1] "artery_diameters_wall"               "glom_bowman_sizes"                  
## [3] "glom_diameters"                      "glom_distance_to_closest_glom"      
## [5] "glom_sizes"                          "glom_tuft_shape_circularity"        
## [7] "glom_tuft_sizes"                     "tubule_diameters"                   
## [9] "tubule_distance_to_closest_instance"
```

subgroups?

```
## [1] "Subgroup analysis is disabled."
```

data missingness:

```
## [1] "Warning: input vectors of unequal length - only complete rows / complete data pairs can be analysed for correlation. 208779 rows with missing data excluded."
```

correlation data:

|  | artery\_diameters\_wall | glom\_bowman\_sizes | glom\_diameters | glom\_distance\_to\_closest\_glom | glom\_sizes | glom\_tuft\_shape\_circularity | glom\_tuft\_sizes | tubule\_diameters | tubule\_distance\_to\_closest\_instance |
| --- | --- | --- | --- | --- | --- | --- | --- | --- | --- |
| artery\_diameters\_wall | 1.00 | 0.01 | 0.00 | 0.03 | 0.01 | 0.00 | 0.01 | 0.01 | -0.01 |
| glom\_bowman\_sizes | 0.01 | 1.00 | 0.59 | -0.01 | 0.71 | -0.27 | 0.46 | 0.01 | -0.02 |
| glom\_diameters | 0.00 | 0.59 | 1.00 | -0.09 | 0.93 | 0.15 | 0.91 | 0.02 | -0.03 |
| glom\_distance\_to\_closest\_glom | 0.03 | -0.01 | -0.09 | 1.00 | -0.06 | -0.06 | -0.06 | 0.00 | -0.02 |
| glom\_sizes | 0.01 | 0.71 | 0.93 | -0.06 | 1.00 | -0.03 | 0.95 | 0.02 | -0.03 |
| glom\_tuft\_shape\_circularity | 0.00 | -0.27 | 0.15 | -0.06 | -0.03 | 1.00 | 0.08 | 0.00 | 0.02 |
| glom\_tuft\_sizes | 0.01 | 0.46 | 0.91 | -0.06 | 0.95 | 0.08 | 1.00 | 0.02 | -0.03 |
| tubule\_diameters | 0.01 | 0.01 | 0.02 | 0.00 | 0.02 | 0.00 | 0.02 | 1.00 | -0.24 |
| tubule\_distance\_to\_closest\_instance | -0.01 | -0.02 | -0.03 | -0.02 | -0.03 | 0.02 | -0.03 | -0.24 | 1.00 |

correlation plot:
