# Supplementary material for: tRigon: an R package and Shiny App for integrative (path-)omics data analysis
Source: BMC Bioinformatics. 2024 Mar 5;25:98. doi: 10.1186/s12859-024-05721-w (PMC10916305; doi:10.1186/s12859-024-05721-w)
Supplement: Supplementary file 3 — Additional file 3. tRigon session report in html-format for loading data into the application including a detailed description of the loaded data frame. [file 12859_2024_5721_MOESM3_ESM.html]

Session Report - Loading Data


# Session Report - Loading Data


---

```
##  setting  value
##  version  R version 4.2.2 (2022-10-31 ucrt)
##  os       Windows 10 x64 (build 19045)
##  system   x86_64, mingw32
##  ui       RStudio
##  language (EN)
##  collate  German_Germany.1252
##  ctype    German_Germany.1252
##  tz       Europe/Berlin
##  date     2023-10-20
##  rstudio  1.4.1106 Tiger Daylily (desktop)
##  pandoc   2.11.4 @ C:/Program Files/RStudio/bin/pandoc/ (via rmarkdown)
```

file loaded:

```
## [1] "KPMP_processed_data.csv"
```

columns in loaded file:

```
##  [1] "patient_ID"                          "sex"                                
##  [3] "age_strat"                           "type"                               
##  [5] "gfr_strat"                           "proteinuria"                        
##  [7] "hba1c"                               "albuminuria"                        
##  [9] "diabetes"                            "diabetes_years"                     
## [11] "hypertension"                        "hypertension_years"                 
## [13] "raas_blockade"                       "X_tissue_size"                      
## [15] "artery_N"                            "artery_area."                       
## [17] "artery_diameters"                    "artery_diameters_lumen"             
## [19] "artery_diameters_wall"               "artery_sizes"                       
## [21] "artery_sizes_lumen"                  "artery_sizes_wall"                  
## [23] "artery_structure_distance"           "glom_N"                             
## [25] "glom_area."                          "glom_bowman_sizes"                  
## [27] "glom_diameters"                      "glom_distance_to_closest_glom"      
## [29] "glom_shape_circularity"              "glom_shape_eccentricity"            
## [31] "glom_shape_elongation"               "glom_shape_solidity"                
## [33] "glom_sizes"                          "glom_tuft_shape_circularity"        
## [35] "glom_tuft_shape_eccentricity"        "glom_tuft_shape_elongation"         
## [37] "glom_tuft_shape_solidity"            "glom_tuft_sizes"                    
## [39] "interstitium_area."                  "lumen_N_segments"                   
## [41] "lumen_area."                         "tubule_N"                           
## [43] "tubule_area."                        "tubule_diameters"                   
## [45] "tubule_distance_to_closest_instance" "tubule_sizes"                       
## [47] "tuft_N_segments"                     "tuft_area."
```

n columns in loaded file:

```
## [1] 48
```

n rows in loaded file:

```
## [1] 211287
```
