# Supplementary material for: tRigon: an R package and Shiny App for integrative (path-)omics data analysis
Source: BMC Bioinformatics. 2024 Mar 5;25:98. doi: 10.1186/s12859-024-05721-w (PMC10916305; doi:10.1186/s12859-024-05721-w)
Supplement: Supplementary file 4 — Additional file 4. tRigon session report in html-format for processing omics datasets including a detailed description of input files, processing settings and the processed data frame. [file 12859_2024_5721_MOESM4_ESM.html]

Session Report - Data Processing


# Session Report - Data Processing


---

```
##  setting  value
##  version  R version 4.2.2 (2022-10-31 ucrt)
##  os       Windows 10 x64 (build 19045)
##  system   x86_64, mingw32
##  ui       RStudio
##  language (EN)
##  collate  German_Germany.1252
##  ctype    German_Germany.1252
##  tz       Europe/Berlin
##  date     2023-10-20
##  rstudio  1.4.1106 Tiger Daylily (desktop)
##  pandoc   2.11.4 @ C:/Program Files/RStudio/bin/pandoc/ (via rmarkdown)
```

data input:

```
## [1] "Human data was processed."
```

n feature files processed:

```
## [1] 174
```

feature files processed:

```
##   [1] "0c9a0630-f01c-4021-b4ba-6d1615a02e10-S-1908-010066_1919_PAS_1of2_1_features.csv"
##   [2] "0c9a0630-f01c-4021-b4ba-6d1615a02e10-S-1908-010066_1919_PAS_1of2_2_features.csv"
##   [3] "0e481520-ae9e-4616-a0b2-27e1f1a31955_18-142_PAS_4of6_1_features.csv"            
##   [4] "2d2bfed9-0ae2-4f66-bcc8-7f86363b58c3-S-1908-009782_1915_PAS_1of2_1_features.csv"
##   [5] "2d2bfed9-0ae2-4f66-bcc8-7f86363b58c3-S-1908-009782_1915_PAS_1of2_2_features.csv"
##   [6] "2fda166d-a6d0-46a7-acfa-124e0e51dce2-S-2001-019413_1927_PAS_1of2_1_features.csv"
##   [7] "2fda166d-a6d0-46a7-acfa-124e0e51dce2-S-2001-019413_1927_PAS_1of2_2_features.csv"
##   [8] "3d90fa26-09eb-425f-b3bc-5116bff0e7af-S-2006-002185_1928_PAS_2of2_1_features.csv"
##   [9] "3d90fa26-09eb-425f-b3bc-5116bff0e7af-S-2006-002185_1928_PAS_2of2_2_features.csv"
##  [10] "3daf2299-1c81-4a33-9596-0acd09e340e7-S-1909-007149_1909_PAS_1of2_1_features.csv"
##  [11] "3daf2299-1c81-4a33-9596-0acd09e340e7-S-1909-007149_1909_PAS_1of2_2_features.csv"
##  [12] "3daf2299-1c81-4a33-9596-0acd09e340e7-S-1909-007149_1909_PAS_1of2_3_features.csv"
##  [13] "3daf2299-1c81-4a33-9596-0acd09e340e7-S-1909-007149_1909_PAS_1of2_4_features.csv"
##  [14] "4b2f93d1-2928-4f3d-a907-e099afb7a1b9-S-1905-017549_1907_PAS_2of2_1_features.csv"
##  [15] "4b2f93d1-2928-4f3d-a907-e099afb7a1b9-S-1905-017549_1907_PAS_2of2_2_features.csv"
##  [16] "4d67cb77-12cd-49d4-b3bd-6f465fa68437-S-2006-002186_1928_PAS_1of2_1_features.csv"
##  [17] "4d67cb77-12cd-49d4-b3bd-6f465fa68437-S-2006-002186_1928_PAS_1of2_2_features.csv"
##  [18] "4e830c09-7764-407b-834d-e4ad19c4b3be-S-1910-000136_1922_PAS_2of2_1_features.csv"
##  [19] "4e830c09-7764-407b-834d-e4ad19c4b3be-S-1910-000136_1922_PAS_2of2_2_features.csv"
##  [20] "5d07f47b-dd32-4c09-92ea-fd084f4ca2e8_18-162_PAS_1of6_1_features.csv"            
##  [21] "5d723ec6-3cfd-4a2d-b84e-47400bfd163f-S-2001-000137_1925_PAS_1of2_1_features.csv"
##  [22] "5d723ec6-3cfd-4a2d-b84e-47400bfd163f-S-2001-000137_1925_PAS_1of2_2_features.csv"
##  [23] "5d723ec6-3cfd-4a2d-b84e-47400bfd163f-S-2001-000137_1925_PAS_1of2_3_features.csv"
##  [24] "5d723ec6-3cfd-4a2d-b84e-47400bfd163f-S-2001-000137_1925_PAS_1of2_4_features.csv"
##  [25] "6bcb7c47-1351-4eff-b5d2-4042c6682255-S-2006-002245_1929_PAS_1of2_1_features.csv"
##  [26] "6bcb7c47-1351-4eff-b5d2-4042c6682255-S-2006-002245_1929_PAS_1of2_2_features.csv"
##  [27] "6bcb7c47-1351-4eff-b5d2-4042c6682255-S-2006-002245_1929_PAS_1of2_3_features.csv"
##  [28] "6bcb7c47-1351-4eff-b5d2-4042c6682255-S-2006-002245_1929_PAS_1of2_4_features.csv"
##  [29] "6cc06bbe-c32f-46ea-bb83-d9a4678ec597-S-1910-000090_1921_PAS_1of2_1_features.csv"
##  [30] "6cc06bbe-c32f-46ea-bb83-d9a4678ec597-S-1910-000090_1921_PAS_1of2_2_features.csv"
##  [31] "6cc06bbe-c32f-46ea-bb83-d9a4678ec597-S-1910-000090_1921_PAS_1of2_3_features.csv"
##  [32] "6cc06bbe-c32f-46ea-bb83-d9a4678ec597-S-1910-000090_1921_PAS_1of2_4_features.csv"
##  [33] "6cc06bbe-c32f-46ea-bb83-d9a4678ec597-S-1910-000090_1921_PAS_1of2_5_features.csv"
##  [34] "6cc06bbe-c32f-46ea-bb83-d9a4678ec597-S-1910-000090_1921_PAS_1of2_6_features.csv"
##  [35] "7daf471c-957e-4ebf-a206-bc0fef11cf2f_18-162_PAS_4of6_1_features.csv"            
##  [36] "7f3d65c3-b7df-4f26-b7da-074ae184868e-S-2006-002244_1929_PAS_2of2_1_features.csv"
##  [37] "7f3d65c3-b7df-4f26-b7da-074ae184868e-S-2006-002244_1929_PAS_2of2_2_features.csv"
##  [38] "9b037714-5f3f-4946-9619-b7975b59bfe6-S-1908-009641_1913_PAS_1of2_1_features.csv"
##  [39] "12ad1312-b563-45e8-9fe3-2c1b559d7674_18-142_PAS_1of6_1_features.csv"            
##  [40] "16dc8e6f-524c-48cb-9252-46140fd74090_18-162_PAS_6of6_1_features.csv"            
##  [41] "20c3ec3e-1f0e-45ab-b029-ddb259b76b2f_18-142_PAS_5of6_1_features.csv"            
##  [42] "20c19df9-4064-4675-9fff-364431b436bb-31_10001_PAS_1of2_1_features.csv"          
##  [43] "20c19df9-4064-4675-9fff-364431b436bb-31_10001_PAS_1of2_2_features.csv"          
##  [44] "20c19df9-4064-4675-9fff-364431b436bb-31_10001_PAS_1of2_3_features.csv"          
##  [45] "28c56ff4-0131-4154-817d-3ee04357f8c7-S-1905-018731_1901_PAS_2of2_1_features.csv"
##  [46] "28c56ff4-0131-4154-817d-3ee04357f8c7-S-1905-018731_1901_PAS_2of2_2_features.csv"
##  [47] "46d62a85-3527-402b-b3e8-d504242d545c-S-1908-009781_1915_PAS_2of2_1_features.csv"
##  [48] "46d62a85-3527-402b-b3e8-d504242d545c-S-1908-009781_1915_PAS_2of2_2_features.csv"
##  [49] "48d69e1d-a359-4661-bb34-29fb78e08726-S-1908-009688_1914_PAS_1of2_1_features.csv"
##  [50] "53c6c2a0-d8c5-43c7-a516-508a99ee0492-S-2006-004764_1930_PAS_1of2_1_features.csv"
##  [51] "53c6c2a0-d8c5-43c7-a516-508a99ee0492-S-2006-004764_1930_PAS_1of2_2_features.csv"
##  [52] "59eaa15d-a1d5-4990-93bc-7b53b590fab5-S-2006-004670_2006_PAS_1of2_1_features.csv"
##  [53] "59eaa15d-a1d5-4990-93bc-7b53b590fab5-S-2006-004670_2006_PAS_1of2_2_features.csv"
##  [54] "59eaa15d-a1d5-4990-93bc-7b53b590fab5-S-2006-004670_2006_PAS_1of2_3_features.csv"
##  [55] "060e75ad-0819-4007-b5bc-18a39dbcefdd-S-2001-019412_1927_PAS_2of2_1_features.csv"
##  [56] "060e75ad-0819-4007-b5bc-18a39dbcefdd-S-2001-019412_1927_PAS_2of2_2_features.csv"
##  [57] "61a1c11d-d869-4868-ba9b-5f31fbd1dbff_18-162_PAS_3of6_1_features.csv"            
##  [58] "62c5bb5f-01a6-4a93-bdc0-12c7c36a5fe2-S-1905-018684_1906_PAS_2of2_1_features.csv"
##  [59] "62c5bb5f-01a6-4a93-bdc0-12c7c36a5fe2-S-1905-018684_1906_PAS_2of2_2_features.csv"
##  [60] "64e4690a-70de-4b4c-b39a-b909f050ca5e-S-1908-000846_1917_PAS_1of2_1_features.csv"
##  [61] "64e4690a-70de-4b4c-b39a-b909f050ca5e-S-1908-000846_1917_PAS_1of2_2_features.csv"
##  [62] "71dcec3d-8e05-4860-a79d-594c040b9c90-S-1908-009640_1913_PAS_2of2_1_features.csv"
##  [63] "72d1aa74-ae21-4c4a-8b96-c716761d6cdb-S-1905-018685_1906_PAS_1of2_1_features.csv"
##  [64] "72d1aa74-ae21-4c4a-8b96-c716761d6cdb-S-1905-018685_1906_PAS_1of2_2_features.csv"
##  [65] "81ccdeb8-3be8-4599-93da-37db3d7350cf-S-1904-008128_1902_PAS_2of2_1_features.csv"
##  [66] "230ae9a5-1728-4bda-a885-c7e161666b25-S-1905-017596_1905_PAS_2of2_1_features.csv"
##  [67] "230ae9a5-1728-4bda-a885-c7e161666b25-S-1905-017596_1905_PAS_2of2_2_features.csv"
##  [68] "316b1c40-15cf-437d-8061-87af293a1b67-S-1905-018732_1901_PAS_1of2_1_features.csv"
##  [69] "316b1c40-15cf-437d-8061-87af293a1b67-S-1905-018732_1901_PAS_1of2_2_features.csv"
##  [70] "338f60cc-f99c-42eb-980e-89425e2fc071-S-2001-000090_1923_PAS_1of2_1_features.csv"
##  [71] "338f60cc-f99c-42eb-980e-89425e2fc071-S-2001-000090_1923_PAS_1of2_2_features.csv"
##  [72] "338f60cc-f99c-42eb-980e-89425e2fc071-S-2001-000090_1923_PAS_1of2_3_features.csv"
##  [73] "338f60cc-f99c-42eb-980e-89425e2fc071-S-2001-000090_1923_PAS_1of2_4_features.csv"
##  [74] "502d2911-4815-44e0-b66f-b1910e8808e9-S-1910-000043_1920_PAS_1of2_1_features.csv"
##  [75] "502d2911-4815-44e0-b66f-b1910e8808e9-S-1910-000043_1920_PAS_1of2_2_features.csv"
##  [76] "502d2911-4815-44e0-b66f-b1910e8808e9-S-1910-000043_1920_PAS_1of2_3_features.csv"
##  [77] "502d2911-4815-44e0-b66f-b1910e8808e9-S-1910-000043_1920_PAS_1of2_4_features.csv"
##  [78] "502d2911-4815-44e0-b66f-b1910e8808e9-S-1910-000043_1920_PAS_1of2_5_features.csv"
##  [79] "502d2911-4815-44e0-b66f-b1910e8808e9-S-1910-000043_1920_PAS_1of2_6_features.csv"
##  [80] "674c9635-5714-40a4-94f7-df1710f4c072-S-1903-005466_1903_PAS_1of2_1_features.csv"
##  [81] "674c9635-5714-40a4-94f7-df1710f4c072-S-1903-005466_1903_PAS_1of2_2_features.csv"
##  [82] "976f3c99-c21d-4143-828c-c32470a0ea7a-S-2002-007742_2002_PAS_2of2_1_features.csv"
##  [83] "976f3c99-c21d-4143-828c-c32470a0ea7a-S-2002-007742_2002_PAS_2of2_2_features.csv"
##  [84] "1304fde6-ad62-450f-a594-543c197278b6-S-1910-000184_1910_PAS_1of2_1_features.csv"
##  [85] "1304fde6-ad62-450f-a594-543c197278b6-S-1910-000184_1910_PAS_1of2_2_features.csv"
##  [86] "5926e109-f49f-42a7-8b5b-f97381ae026b_18-142_PAS_2of6_1_features.csv"            
##  [87] "7586b5d6-f1f3-4e2e-af7e-67e1f8703d31-S-1909-007148_1909_PAS_2of2_1_features.csv"
##  [88] "7586b5d6-f1f3-4e2e-af7e-67e1f8703d31-S-1909-007148_1909_PAS_2of2_2_features.csv"
##  [89] "14196f17-d87e-41b7-b452-4059cf673d0c_322_PAS_1of2_1_features.csv"               
##  [90] "14196f17-d87e-41b7-b452-4059cf673d0c_322_PAS_1of2_2_features.csv"               
##  [91] "19374c21-8261-48d3-adee-98f4ff52d8da-S-1908-010065_1919_PAS_2of2_1_features.csv"
##  [92] "19374c21-8261-48d3-adee-98f4ff52d8da-S-1908-010065_1919_PAS_2of2_2_features.csv"
##  [93] "627425fd-5092-4c6f-b17e-70d891539a44-S-2002-007743_2002_PAS_1of2_1_features.csv"
##  [94] "627425fd-5092-4c6f-b17e-70d891539a44-S-2002-007743_2002_PAS_1of2_2_features.csv"
##  [95] "760649cb-be20-4559-8f29-5fb4a3225e43_18-162_PAS_5of6_1_features.csv"            
##  [96] "829067a5-eda4-415d-8e2e-fa9a15ac583c_18-162_PAS_2of6_1_features.csv"            
##  [97] "a3d7c720-51e1-4c9f-a528-7bc6b13cd3e4-S-1905-017550_1907_PAS_1of2_1_features.csv"
##  [98] "a3d7c720-51e1-4c9f-a528-7bc6b13cd3e4-S-1905-017550_1907_PAS_1of2_2_features.csv"
##  [99] "a3d7c720-51e1-4c9f-a528-7bc6b13cd3e4-S-1905-017550_1907_PAS_1of2_3_features.csv"
## [100] "a3d7c720-51e1-4c9f-a528-7bc6b13cd3e4-S-1905-017550_1907_PAS_1of2_4_features.csv"
## [101] "a9d5e20a-a4dd-4d70-a0fb-7b770352fdf7_18-142_PAS_3of6_1_features.csv"            
## [102] "a070acf9-479a-46e5-959d-472091f19f68-S-1908-000752_1912_PAS_1of2_1_features.csv"
## [103] "a070acf9-479a-46e5-959d-472091f19f68-S-1908-000752_1912_PAS_1of2_2_features.csv"
## [104] "a070acf9-479a-46e5-959d-472091f19f68-S-1908-000752_1912_PAS_1of2_3_features.csv"
## [105] "a070acf9-479a-46e5-959d-472091f19f68-S-1908-000752_1912_PAS_1of2_4_features.csv"
## [106] "a070acf9-479a-46e5-959d-472091f19f68-S-1908-000752_1912_PAS_1of2_5_features.csv"
## [107] "a070acf9-479a-46e5-959d-472091f19f68-S-1908-000752_1912_PAS_1of2_6_features.csv"
## [108] "a71eff4a-4751-423f-91aa-c273ea6c08cf-S-2001-005357_2001_PAS_1of2_1_features.csv"
## [109] "a71eff4a-4751-423f-91aa-c273ea6c08cf-S-2001-005357_2001_PAS_1of2_2_features.csv"
## [110] "a88491ba-ad1b-4aa2-9465-edab2eb9f8e9-31_10001_PAS_2of2_1_features.csv"          
## [111] "a88491ba-ad1b-4aa2-9465-edab2eb9f8e9-31_10001_PAS_2of2_2_features.csv"          
## [112] "aa008e8f-e27f-4157-b765-069f3d106654-S-1910-000183_1910_PAS_2of2_1_features.csv"
## [113] "aa008e8f-e27f-4157-b765-069f3d106654-S-1910-000183_1910_PAS_2of2_2_features.csv"
## [114] "b9ba5d75-0d13-4b1b-94fc-22f4b3f8b99b-S-1910-000137_1922_PAS_1of2_1_features.csv"
## [115] "b9ba5d75-0d13-4b1b-94fc-22f4b3f8b99b-S-1910-000137_1922_PAS_1of2_2_features.csv"
## [116] "b9ba5d75-0d13-4b1b-94fc-22f4b3f8b99b-S-1910-000137_1922_PAS_1of2_3_features.csv"
## [117] "b9ba5d75-0d13-4b1b-94fc-22f4b3f8b99b-S-1910-000137_1922_PAS_1of2_4_features.csv"
## [118] "ba7e6432-05a0-4e3f-87e9-cfd3dcbd30a0-S-1904-007293_1904_PAS_1of2_1_features.csv"
## [119] "ba7e6432-05a0-4e3f-87e9-cfd3dcbd30a0-S-1904-007293_1904_PAS_1of2_2_features.csv"
## [120] "bac31e5e-6ef2-4388-87f8-87bd5c393654-S-1908-009687_1914_PAS_2of2_1_features.csv"
## [121] "c35c107f-0d8e-40a2-bd84-f78795ed458d-S-2001-005404_1926_PAS_1of2_1_features.csv"
## [122] "c35c107f-0d8e-40a2-bd84-f78795ed458d-S-2001-005404_1926_PAS_1of2_2_features.csv"
## [123] "c99e8e27-9990-4cd8-933c-b3bbade3538d-S-1908-009831_1908_PAS_1of2_1_features.csv"
## [124] "c228b00f-66fa-4e50-a860-ad7c51c13d23-S-1903-005465_1903_PAS_2of2_1_features.csv"
## [125] "c228b00f-66fa-4e50-a860-ad7c51c13d23-S-1903-005465_1903_PAS_2of2_2_features.csv"
## [126] "cbbccee2-d96d-4b5d-a1f3-3f3ff60c07d2-S-1910-000089_1921_PAS_2of2_1_features.csv"
## [127] "cbbccee2-d96d-4b5d-a1f3-3f3ff60c07d2-S-1910-000089_1921_PAS_2of2_2_features.csv"
## [128] "cbe81657-14aa-42a1-bec5-aec878b80596-S-1908-009830_1908_PAS_2of2_1_features.csv"
## [129] "cc9aa83b-0cc7-4ed4-88b4-aba067f07095-S-1908-000892_1916_PAS_1of2_1_features.csv"
## [130] "cc9aa83b-0cc7-4ed4-88b4-aba067f07095-S-1908-000892_1916_PAS_1of2_2_features.csv"
## [131] "d6c378ab-255a-4f41-a0c0-33cd7d873f73-S-1905-017597_1905_PAS_1of2_1_features.csv"
## [132] "d6c378ab-255a-4f41-a0c0-33cd7d873f73-S-1905-017597_1905_PAS_1of2_2_features.csv"
## [133] "d6c378ab-255a-4f41-a0c0-33cd7d873f73-S-1905-017597_1905_PAS_1of2_3_features.csv"
## [134] "d6c378ab-255a-4f41-a0c0-33cd7d873f73-S-1905-017597_1905_PAS_1of2_4_features.csv"
## [135] "d9e4f663-69dc-4a0c-bfe0-47e085aae04e_18-142_PAS_6of6_1_features.csv"            
## [136] "d49b022c-7399-4a37-bb7e-f0079acd044d-S-2001-000136_1925_PAS_2of2_1_features.csv"
## [137] "d49b022c-7399-4a37-bb7e-f0079acd044d-S-2001-000136_1925_PAS_2of2_2_features.csv"
## [138] "d49b022c-7399-4a37-bb7e-f0079acd044d-S-2001-000136_1925_PAS_2of2_3_features.csv"
## [139] "d8655d07-08d8-4c45-9a06-58e4b6bba4cf-S-1908-000799_1918_PAS_1of2_1_features.csv"
## [140] "dae8b109-2968-4596-9c56-3754185b7cf9-S-1908-000845_1917_PAS_2of2_1_features.csv"
## [141] "dae8b109-2968-4596-9c56-3754185b7cf9-S-1908-000845_1917_PAS_2of2_2_features.csv"
## [142] "dbdcd9a2-62a7-4988-86a2-90cf1ea57242-S-1910-000042_1920_PAS_2of2_1_features.csv"
## [143] "dbdcd9a2-62a7-4988-86a2-90cf1ea57242-S-1910-000042_1920_PAS_2of2_2_features.csv"
## [144] "dbdcd9a2-62a7-4988-86a2-90cf1ea57242-S-1910-000042_1920_PAS_2of2_3_features.csv"
## [145] "e6b3aad7-3d6b-4072-accc-65464bdf590b-S-1904-007292_1904_PAS_2of2_1_features.csv"
## [146] "e6b3aad7-3d6b-4072-accc-65464bdf590b-S-1904-007292_1904_PAS_2of2_2_features.csv"
## [147] "e6c166fd-0b8b-4dbe-8fea-7fd3310d9243-S-2001-005356_2001_PAS_2of2_1_features.csv"
## [148] "e6c166fd-0b8b-4dbe-8fea-7fd3310d9243-S-2001-005356_2001_PAS_2of2_2_features.csv"
## [149] "ed4e6ba3-a00c-4240-854c-c218b1db6722-S-2001-005403_1926_PAS_2of2_1_features.csv"
## [150] "ed4e6ba3-a00c-4240-854c-c218b1db6722-S-2001-005403_1926_PAS_2of2_2_features.csv"
## [151] "f0b19ce6-0b3c-4a0c-8f05-f607c478a6f0-S-2001-000043_1924_PAS_1of2_1_features.csv"
## [152] "f0b19ce6-0b3c-4a0c-8f05-f607c478a6f0-S-2001-000043_1924_PAS_1of2_2_features.csv"
## [153] "f0b19ce6-0b3c-4a0c-8f05-f607c478a6f0-S-2001-000043_1924_PAS_1of2_3_features.csv"
## [154] "f0b19ce6-0b3c-4a0c-8f05-f607c478a6f0-S-2001-000043_1924_PAS_1of2_4_features.csv"
## [155] "f1f7bccc-feb9-42fe-a76c-8a488e6b1986-S-2006-004669_2006_PAS_2of2_1_features.csv"
## [156] "f1f7bccc-feb9-42fe-a76c-8a488e6b1986-S-2006-004669_2006_PAS_2of2_2_features.csv"
## [157] "f4f4e11f-5ecc-4ce7-a813-3123facd7d50-S-2006-004763_1930_PAS_2of2_1_features.csv"
## [158] "f4f4e11f-5ecc-4ce7-a813-3123facd7d50-S-2006-004763_1930_PAS_2of2_2_features.csv"
## [159] "f6b1826e-5a17-473f-b541-94a96e7bd703_322_PAS_2of2_1_features.csv"               
## [160] "f6b1826e-5a17-473f-b541-94a96e7bd703_322_PAS_2of2_2_features.csv"               
## [161] "f7da827d-dafc-472e-9c26-f26f5cd713d9-S-1908-000751_1912_PAS_2of2_1_features.csv"
## [162] "f7da827d-dafc-472e-9c26-f26f5cd713d9-S-1908-000751_1912_PAS_2of2_2_features.csv"
## [163] "f7da827d-dafc-472e-9c26-f26f5cd713d9-S-1908-000751_1912_PAS_2of2_3_features.csv"
## [164] "f7da827d-dafc-472e-9c26-f26f5cd713d9-S-1908-000751_1912_PAS_2of2_4_features.csv"
## [165] "f7da827d-dafc-472e-9c26-f26f5cd713d9-S-1908-000751_1912_PAS_2of2_5_features.csv"
## [166] "f7da827d-dafc-472e-9c26-f26f5cd713d9-S-1908-000751_1912_PAS_2of2_6_features.csv"
## [167] "f51be06b-d660-4358-abe5-f94c1647c07e-S-1908-000893_1916_PAS_2of2_1_features.csv"
## [168] "f51be06b-d660-4358-abe5-f94c1647c07e-S-1908-000893_1916_PAS_2of2_2_features.csv"
## [169] "f51be06b-d660-4358-abe5-f94c1647c07e-S-1908-000893_1916_PAS_2of2_3_features.csv"
## [170] "fad66413-d6dd-4915-a151-2b64c51978d9-S-2001-000089_1923_PAS_2of2_1_features.csv"
## [171] "fad66413-d6dd-4915-a151-2b64c51978d9-S-2001-000089_1923_PAS_2of2_2_features.csv"
## [172] "fba824af-8ea2-4ec6-8159-e95fdb65c637-S-1904-008129_1902_PAS_1of2_1_features.csv"
## [173] "fea35cdd-95d1-4d61-ac8a-f3a6a4c4566a-S-2001-000042_1924_PAS_2of2_1_features.csv"
## [174] "fea35cdd-95d1-4d61-ac8a-f3a6a4c4566a-S-2001-000042_1924_PAS_2of2_2_features.csv"
```

metadata file processed:

```
## [1] "KPMP_metadata.xlsx"
```

columns in metadata file:

```
##  [1] "pseudonym"          "sex"                "age_strat"          "type"               "gfr_strat"         
##  [6] "proteinuria"        "hba1c"              "albuminuria"        "diabetes"           "diabetes_years"    
## [11] "hypertension"       "hypertension_years" "raas_blockade"
```

columns in processed file:

```
##  [1] "patient_ID"                          "sex"                                
##  [3] "age_strat"                           "type"                               
##  [5] "gfr_strat"                           "proteinuria"                        
##  [7] "hba1c"                               "albuminuria"                        
##  [9] "diabetes"                            "diabetes_years"                     
## [11] "hypertension"                        "hypertension_years"                 
## [13] "raas_blockade"                       "X_tissue_size"                      
## [15] "artery_N"                            "artery_area."                       
## [17] "artery_diameters"                    "artery_diameters_lumen"             
## [19] "artery_diameters_wall"               "artery_sizes"                       
## [21] "artery_sizes_lumen"                  "artery_sizes_wall"                  
## [23] "artery_structure_distance"           "glom_N"                             
## [25] "glom_area."                          "glom_bowman_sizes"                  
## [27] "glom_diameters"                      "glom_distance_to_closest_glom"      
## [29] "glom_shape_circularity"              "glom_shape_eccentricity"            
## [31] "glom_shape_elongation"               "glom_shape_solidity"                
## [33] "glom_sizes"                          "glom_tuft_shape_circularity"        
## [35] "glom_tuft_shape_eccentricity"        "glom_tuft_shape_elongation"         
## [37] "glom_tuft_shape_solidity"            "glom_tuft_sizes"                    
## [39] "interstitium_area."                  "lumen_N_segments"                   
## [41] "lumen_area."                         "tubule_N"                           
## [43] "tubule_area."                        "tubule_diameters"                   
## [45] "tubule_distance_to_closest_instance" "tubule_sizes"                       
## [47] "tuft_N_segments"                     "tuft_area."
```

feature calculations:

```
## [1] "calculations disabled: deleting calculated columns from processed data."
```

n columns in processed file:

```
## [1] 48
```

n rows in processed file:

```
## [1] 211287
```

matched IDs in feature & clinical files:

```
## [1] "All IDs matched!"
```

```
##    patient_ID match
## 1        1925  TRUE
## 2        1924  TRUE
## 3        1929  TRUE
## 4        1927  TRUE
## 5        1930  TRUE
## 6        1928  TRUE
## 7        1906  TRUE
## 8        1923  TRUE
## 9        1921  TRUE
## 10       1909  TRUE
## 11       1905  TRUE
## 12       1926  TRUE
## 13       2001  TRUE
## 14       1912  TRUE
## 15       1914  TRUE
## 16       1915  TRUE
## 17       2002  TRUE
## 18       1916  TRUE
## 19       1920  TRUE
## 20      10001  TRUE
## 21       1918  TRUE
## 22       2006  TRUE
## 23       1901  TRUE
## 24       1917  TRUE
## 25       1922  TRUE
## 26       1910  TRUE
## 27       1913  TRUE
## 28       1904  TRUE
## 29       1919  TRUE
## 30        322  TRUE
## 31       1908  TRUE
## 32       1902  TRUE
## 33       1903  TRUE
## 34       1907  TRUE
## 35     18-142  TRUE
## 36     18-162  TRUE
```
