# Supplementary material for: tRigon: an R package and Shiny App for integrative (path-)omics data analysis
Source: BMC Bioinformatics. 2024 Mar 5;25:98. doi: 10.1186/s12859-024-05721-w (PMC10916305; doi:10.1186/s12859-024-05721-w)
Supplement: Supplementary file 5 — Additional file 5. tRigon session report in html-format for descriptive statistics including all inputs, setting options and outputs. [file 12859_2024_5721_MOESM5_ESM.html]

Session Report - Descriptive Statistics


# Session Report - Descriptive Statistics


---

```
##  setting  value
##  version  R version 4.2.2 (2022-10-31 ucrt)
##  os       Windows 10 x64 (build 19045)
##  system   x86_64, mingw32
##  ui       RStudio
##  language (EN)
##  collate  German_Germany.1252
##  ctype    German_Germany.1252
##  tz       Europe/Berlin
##  date     2023-10-20
##  rstudio  1.4.1106 Tiger Daylily (desktop)
##  pandoc   2.11.4 @ C:/Program Files/RStudio/bin/pandoc/ (via rmarkdown)
```

feature:

```
## [1] "glom_tuft_sizes"
```

group variable:

```
## [1] "gfr_strat"
```

groups:

```
##  [1] "40-49"   "NA"      "50-59"   "70-79"   "30-39"   "80-89"   "60-69"   "110-119" "20-29"   "120-129" "100-109"
```

descriptive statistics:

| gfr\_strat | feature | median | mean | var | sd | min | max | Q1 | Q3 | iqr | n\_datapoints |
| --- | --- | --- | --- | --- | --- | --- | --- | --- | --- | --- | --- |
| 100-109 | glom\_tuft\_sizes | 12763.27 | 14637.78 | 128140871 | 11319.93 | 0 | 55374.44 | 5538.76 | 22610.42 | 17071.65 | 118 |
| 110-119 | glom\_tuft\_sizes | 13801.85 | 15158.15 | 224929017 | 14997.63 | 0 | 85208.91 | 0.00 | 25209.07 | 25209.07 | 299 |
| 120-129 | glom\_tuft\_sizes | 11492.62 | 13477.27 | 147841774 | 12159.02 | 0 | 37051.68 | 248.46 | 25303.51 | 25055.05 | 63 |
| 20-29 | glom\_tuft\_sizes | 2502.66 | 8430.01 | 103848363 | 10190.60 | 0 | 29107.41 | 0.00 | 17392.26 | 17392.26 | 117 |
| 30-39 | glom\_tuft\_sizes | 3869.91 | 8848.45 | 116779589 | 10806.46 | 0 | 48695.53 | 0.00 | 14963.18 | 14963.18 | 497 |
| 40-49 | glom\_tuft\_sizes | 8273.41 | 11524.93 | 173598331 | 13175.67 | 0 | 97279.29 | 0.00 | 18979.32 | 18979.32 | 508 |
| 50-59 | glom\_tuft\_sizes | 4313.16 | 8995.40 | 132890428 | 11527.81 | 0 | 60735.21 | 0.00 | 13915.24 | 13915.24 | 222 |
| 60-69 | glom\_tuft\_sizes | 8457.23 | 15094.18 | 319813896 | 17883.34 | 0 | 63495.31 | 2049.86 | 23081.26 | 21031.40 | 64 |
| 70-79 | glom\_tuft\_sizes | 7150.07 | 9009.57 | 93015696 | 9644.46 | 0 | 53451.25 | 0.00 | 15423.99 | 15423.99 | 196 |
| 80-89 | glom\_tuft\_sizes | 9858.25 | 13165.94 | 162558043 | 12749.83 | 0 | 52347.01 | 1472.78 | 25635.14 | 24162.36 | 175 |
| NA | glom\_tuft\_sizes | 13087.12 | 13611.44 | 121308118 | 11014.00 | 0 | 51073.22 | 2677.60 | 20997.46 | 18319.85 | 1062 |
