# Supplementary material for: tRigon: an R package and Shiny App for integrative (path-)omics data analysis
Source: BMC Bioinformatics. 2024 Mar 5;25:98. doi: 10.1186/s12859-024-05721-w (PMC10916305; doi:10.1186/s12859-024-05721-w)
Supplement: Supplementary file 6 — Additional file 6. tRigon session report in html-format for a feature importance analysis including all inputs, setting options and outputs. [file 12859_2024_5721_MOESM6_ESM.html]

Session Report - Feature Importance


# Session Report - Feature Importance


---

```
##  setting  value
##  version  R version 4.2.2 (2022-10-31 ucrt)
##  os       Windows 10 x64 (build 19045)
##  system   x86_64, mingw32
##  ui       RStudio
##  language (EN)
##  collate  German_Germany.1252
##  ctype    German_Germany.1252
##  tz       Europe/Berlin
##  date     2023-10-20
##  rstudio  1.4.1106 Tiger Daylily (desktop)
##  pandoc   NA
```

features:

```
##  [1] "artery_diameters_MEDIAN"        "artery_sizes_MEDIAN"            "glom_bowman_sizes_MEDIAN"      
##  [4] "glom_diameters_MEDIAN"          "glom_sizes_MEDIAN"              "glom_tuft_sizes_MEDIAN"        
##  [7] "tubule_diameters_MEDIAN"        "tubule_sizes_MEDIAN"            "tuft_shape_circularity_MEDIAN" 
## [10] "tuft_shape_eccentricity_MEDIAN" "tuft_shape_elongation_MEDIAN"   "tuft_shape_solidity_MEDIAN"
```

dependent variable:

```
## [1] "type"
```

groups / levels of dependent variable:

```
## [1] "CKD"               "Tumor Nephrectomy" "AKI"
```

feature importance method:

```
## [1] "recursive feature elimination (RFE) with 5-fold cross-validation and 5 repeats for classification of groups of the dependent variable. Data is distributed in a random 80/20 split for training and testing."
```

feature imbalance:

```
## [1] "Warning: input vectors of unequal length - only complete rows can be analysed for feature importance. 211136 rows with missing data excluded."
```

feature importance output:

```
## [[1]]
## 
## Recursive feature selection
## 
## Outer resampling method: Cross-Validated (5 fold, repeated 5 times) 
## 
## Resampling performance over subset size:
## 
##  Variables Accuracy  Kappa AccuracySD KappaSD Selected
##         12   0.8347 0.5411    0.05727  0.1638        *
## 
## The top 5 variables (out of 12):
##    tuft_shape_circularity_MEDIAN, tubule_diameters_MEDIAN, tubule_sizes_MEDIAN, glom_tuft_sizes_MEDIAN, tuft_shape_solidity_MEDIAN
## 
## 
## [[2]]
##                                         AKI          CKD Tumor Nephrectomy MeanDecreaseAccuracy MeanDecreaseGini
## tuft_shape_circularity_MEDIAN   0.023970158 0.0326445636       0.187147619         0.0416265569         6.480984
## tubule_diameters_MEDIAN         0.065071812 0.0299912170       0.037533333         0.0365350001         7.212644
## tubule_sizes_MEDIAN             0.040937346 0.0264726079       0.047355556         0.0305889051         6.180842
## glom_tuft_sizes_MEDIAN          0.024341930 0.0181916042       0.092561905         0.0245619806         4.511071
## tuft_shape_solidity_MEDIAN      0.021355307 0.0151867617       0.058923810         0.0189365886         3.294175
## glom_diameters_MEDIAN           0.021896920 0.0150696241       0.049681746         0.0187141758         4.721308
## glom_sizes_MEDIAN               0.016088045 0.0157775686       0.036776190         0.0167549234         3.744875
## tuft_shape_elongation_MEDIAN    0.024674842 0.0080129629       0.037919048         0.0132166144         3.172572
## tuft_shape_eccentricity_MEDIAN  0.013672339 0.0068555313       0.028066667         0.0095820053         3.470092
## glom_bowman_sizes_MEDIAN        0.004369741 0.0045138608       0.013352381         0.0047941512         3.098574
## artery_diameters_MEDIAN         0.006522367 0.0006454696       0.006219048         0.0020744171         2.751681
## artery_sizes_MEDIAN            -0.001300540 0.0012574458       0.007628571         0.0007486779         2.623084
## 
## [[3]]
##  Accuracy     Kappa 
## 0.7931034 0.4081633
```

feature importance plot:
