# Supplementary material for: tRigon: an R package and Shiny App for integrative (path-)omics data analysis
Source: BMC Bioinformatics. 2024 Mar 5;25:98. doi: 10.1186/s12859-024-05721-w (PMC10916305; doi:10.1186/s12859-024-05721-w)
Supplement: Supplementary file 7 — Additional file 7. tRigon session report in html-format for feature plots including all inputs, setting options and outputs. [file 12859_2024_5721_MOESM7_ESM.html]

Session Report - Plots


# Session Report - Plots


---

```
##  setting  value
##  version  R version 4.2.2 (2022-10-31 ucrt)
##  os       Windows 10 x64 (build 19045)
##  system   x86_64, mingw32
##  ui       RStudio
##  language (EN)
##  collate  German_Germany.1252
##  ctype    German_Germany.1252
##  tz       Europe/Berlin
##  date     2023-10-20
##  rstudio  1.4.1106 Tiger Daylily (desktop)
##  pandoc   2.11.4 @ C:/Program Files/RStudio/bin/pandoc/ (via rmarkdown)
```

feature:

```
## [1] "glom_tuft_sizes"
```

group variable:

```
## [1] "type"
```

groups:

```
## [1] "CKD"               "Tumor Nephrectomy" "AKI"
```

selected plot:

```
## [1] "Violin with Boxplot"
```

plot scale:

```
## [1] "Logarithmic-scale is enabled."
```

plot:
