# Supplementary material for: tRigon: an R package and Shiny App for integrative (path-)omics data analysis
Source: BMC Bioinformatics. 2024 Mar 5;25:98. doi: 10.1186/s12859-024-05721-w (PMC10916305; doi:10.1186/s12859-024-05721-w)
Supplement: Supplementary file 8 — Additional file 8. tRigon session report in html-format for statistical testing including all inputs, setting options and outputs. [file 12859_2024_5721_MOESM8_ESM.html]

Session Report - Statistical Tests


# Session Report - Statistical Tests


---

```
##  setting  value
##  version  R version 4.2.2 (2022-10-31 ucrt)
##  os       Windows 10 x64 (build 19045)
##  system   x86_64, mingw32
##  ui       RStudio
##  language (EN)
##  collate  German_Germany.1252
##  ctype    German_Germany.1252
##  tz       Europe/Berlin
##  date     2023-10-20
##  rstudio  1.4.1106 Tiger Daylily (desktop)
##  pandoc   2.11.4 @ C:/Program Files/RStudio/bin/pandoc/ (via rmarkdown)
```

feature:

```
## [1] "glom_tuft_sizes"
```

group variable:

```
## [1] "gfr_strat"
```

groups:

```
##  [1] "40-49"   "NA"      "50-59"   "70-79"   "30-39"   "80-89"   "60-69"   "110-119" "20-29"   "120-129" "100-109"
```

selected statistical test:

```
## [1] "two-sided pairwise Wilcoxon-Rank test with bonferroni-adjustment for multiple testing"
```

output statistical test:

```
## 
##  Pairwise comparisons using Wilcoxon rank sum test with continuity correction 
## 
## data:  df_var[[feature]][complete.cases(df_var[[feature]], df_var[[group_col]])] and df_var[[group_col]][complete.cases(df_var[[group_col]], df_var[[feature]])] 
## 
##         100-109 110-119 120-129 20-29   30-39   40-49   50-59   60-69   70-79   80-89  
## 110-119 1.00000 -       -       -       -       -       -       -       -       -      
## 120-129 1.00000 1.00000 -       -       -       -       -       -       -       -      
## 20-29   2.5e-05 0.00571 0.23289 -       -       -       -       -       -       -      
## 30-39   5.4e-08 2.3e-05 0.33473 1.00000 -       -       -       -       -       -      
## 40-49   0.00708 0.34695 1.00000 0.39106 0.04544 -       -       -       -       -      
## 50-59   1.2e-06 0.00035 0.22142 1.00000 1.00000 0.18772 -       -       -       -      
## 60-69   1.00000 1.00000 1.00000 0.39429 0.37874 1.00000 0.73937 -       -       -      
## 70-79   9.1e-05 0.00300 0.57804 1.00000 1.00000 1.00000 1.00000 1.00000 -       -      
## 80-89   1.00000 1.00000 1.00000 0.00585 0.00056 1.00000 0.00272 1.00000 0.04438 -      
## NA      1.00000 1.00000 1.00000 1.2e-05 < 2e-16 7.3e-05 8.5e-10 1.00000 3.6e-07 1.00000
## 
## P value adjustment method: bonferroni
```

output data frame:

|  | 100-109 | 110-119 | 120-129 | 20-29 | 30-39 | 40-49 | 50-59 | 60-69 | 70-79 | 80-89 |
| --- | --- | --- | --- | --- | --- | --- | --- | --- | --- | --- |
| 110-119 | 1.0000000 | NA | NA | NA | NA | NA | NA | NA | NA | NA |
| 120-129 | 1.0000000 | 1.0000000 | NA | NA | NA | NA | NA | NA | NA | NA |
| 20-29 | 0.0000253 | 0.0057068 | 0.2328932 | NA | NA | NA | NA | NA | NA | NA |
| 30-39 | 0.0000001 | 0.0000227 | 0.3347272 | 1.0000000 | NA | NA | NA | NA | NA | NA |
| 40-49 | 0.0070820 | 0.3469508 | 1.0000000 | 0.3910603 | 0.0454426949332043 | NA | NA | NA | NA | NA |
| 50-59 | 0.0000012 | 0.0003483 | 0.2214161 | 1.0000000 | 1 | 0.1877188 | NA | NA | NA | NA |
| 60-69 | 1.0000000 | 1.0000000 | 1.0000000 | 0.3942917 | 0.378736523776829 | 1.0000000 | 0.7393652 | NA | NA | NA |
| 70-79 | 0.0000914 | 0.0030030 | 0.5780424 | 1.0000000 | 1 | 1.0000000 | 1.0000000 | 1 | NA | NA |
| 80-89 | 1.0000000 | 1.0000000 | 1.0000000 | 0.0058512 | 0.000559462003850758 | 1.0000000 | 0.0027217 | 1 | 0.0443820 | NA |
| NA | 1.0000000 | 1.0000000 | 1.0000000 | 0.0000123 | <2e-16 | 0.0000726 | 0.0000000 | 1 | 0.0000004 | 1 |
