# Supplementary material for: tRigon: an R package and Shiny App for integrative (path-)omics data analysis
Source: BMC Bioinformatics. 2024 Mar 5;25:98. doi: 10.1186/s12859-024-05721-w (PMC10916305; doi:10.1186/s12859-024-05721-w)
Supplement: Supplementary file 9 — Additional file 9. Supplementary Material containing Supplementary Tables S1-S4 and Supplementary Figures S1-S9. [file 12859_2024_5721_MOESM9_ESM.pdf]

Supplementary Material to:
tRigon: an R package and Shiny App for integrative (path-)omics data analysis

Running title: tRigon - Toolbox foR InteGrative (path-)Omics data aNalysis

David L. Hölscher[1], Michael Goedertier[1,2], Barbara M. Klinkhammer[1], Patrick Droste[1,3], Ivan G. Costa[2], Peter Boor[1,3,§], Roman D. Bülow[1,§,*]

1 Institute of Pathology, RWTH Aachen University Clinic, Aachen, Germany
2 Institute for Computational Genomics, RWTH Aachen University Clinic, Aachen, Germany
3 Department of Nephrology and Immunology, RWTH Aachen University Clinic, Aachen, Germany

§ Shared senior authors

* Address correspondence to:
Jun.-Prof. Dr. med. Roman D. Bülow
Pauwelsstraße 30
52074 Aachen
rbuelow@ukaachen.de

Table of Contents

Supplementary Table 1: tRigon exemplary output for summary statistics.

Supplementary Table 2: tRigon exemplary output for the non-parametric pairwise two-sided Wilcoxon-rank test.

Supplementary Table 3: tRigon exemplary output for a Pearson correlation matrix.

Supplementary Table 4. tRigon was used to replicate an analysis from[1] reaching the same results.

Supplementary Figure 1: tRigon exemplary output for a combined violin and boxplot.

Supplementary Figure 2: tRigon exemplary output for a k-means clustering plot.

Supplementary Figure 3: tRigon exemplary output for a feature importance plot.

Supplementary Figure 4: tRigon exemplary output for a correlation heatmap.

Supplementary Figure 5: tRigon based reanalysis of the feature "Chromatin Clumping of Epithelial Nuclei" stratified by tumor grade from[1].

Supplementary Figure 6: tRigon based reanalysis of the feature "Complexity of Epithelial Nuclei Boundary" stratified by tumor grade from[1].

Supplementary Figure 7: tRigon based reanalysis of the feature "Number of Low Grade Nuclei Per Epithelial Nest" stratified by tumor grade from[1].

Supplementary Figure 8: tRigon based reanalysis of the feature "Size of Epithelial Nuclei" stratified by tumor grade from[1].

Supplementary Figure 9: tRigon based reanalysis of the feature "Staining of Epithelial Nuclei" stratified by tumor grade from[1].

Supplementary Table 1. tRigon exemplary output for summary statistics. Based on the stratified estimated glomerular filtration rate (gfr_strat) summary statistics for glomerular tuft sizes were calculated. Calculations are based on the Kidney Precision Medicine Project (KPMP) cohort.
var, variance; sd, standard deviation; min, minimum; max, maximum; Q1, first quantile; Q3, third quantile; iqr, interquartile range; n, number of datapoints.

gfr_strat	feature	median	mean	var	sd	min	max	Q1	Q3	iqr	n
100-109	glom_tuft_sizes	12763.3	14637.8	128140870.7	11319.9	0	55374.4	5538.76	22610.4	17071.7	118
110-119	glom_tuft_sizes	13801.9	15158.2	224929017.4	14997.6	0	85208.9	0	25209.1	25209.1	299
120-129	glom_tuft_sizes	11492.6	13477.3	147841774.3	12159	0	37051.7	248.46	25303.5	25055.1	63
20-29	glom_tuft_sizes	2502.66	8430.01	103848362.5	10190.6	0	29107.4	0	17392.3	17392.3	117
30-39	glom_tuft_sizes	3869.91	8848.45	116779588.5	10806.5	0	48695.5	0	14963.2	14963.2	497
40-49	glom_tuft_sizes	8273.41	11524.9	173598331.1	13175.7	0	97279.3	0	18979.3	18979.3	508
50-59	glom_tuft_sizes	4313.16	8995.4	132890427.6	11527.8	0	60735.2	0	13915.2	13915.2	222
60-69	glom_tuft_sizes	8457.23	15094.2	319813896.4	17883.3	0	63495.3	2049.86	21031.4	21031.4	64
70-79	glom_tuft_sizes	7150.07	9009.57	93015696,.6	9644.46	0	53451.3	0	15424	15424	196
80-89	glom_tuft_sizes	9858.25	13165.9	162558042.9	12749.8	0	52347	1472.78	24162.4	24162.4	175
NA	glom_tuft_sizes	13087.1	13611.4	121308118.3	11014	0	51073.2	2677.6	18319.9	18319.9	1062

Supplementary Table 2. tRigon exemplary output for the non-parametric pairwise two-sided Wilcoxon-rank test. Based on the stratified estimated glomerular filtration rate (gfr_strat) *p*-values based on the glomerular tuft sizes were calculated. Calculations are based on the Kidney Precision Medicine Project (KPMP) cohort.

**gfr_strat**	**100-109**	**110-119**	**120-129**	**20-29**	**30-39**	**40-49**	**50-59**	**60-69**	**70-79**	**80-89**
110-119	1	NA	NA	NA	NA	NA	NA	NA	NA	NA
120-129	1	1	NA	NA	NA	NA	NA	NA	NA	NA
20-29	<0.0001	0.0057	0.2329	NA	NA	NA	NA	NA	NA	NA
30-39	<0.0001	<0.0001	0.3347	1	NA	NA	NA	NA	NA	NA
40-49	0.0071	0.3470	1	0.3911	0.0454	NA	NA	NA	NA	NA
50-59	<0.0001	0.0003	0.2214	1	1	0.1877	NA	NA	NA	NA
60-69	1	1	1	0.3943	0.3787	1	0.7394	NA	NA	NA
70-79	<0.0001	0.0030	0.5780	1	1	1	1	1	NA	NA
80-89	1	1	1	0.0059	0.0006	1	0.0027	1	0.0444	NA
NA	1	1	1	<0.0001	<0.0001	<0.0001	<0.0001	1	<0.0001	1

Supplementary Table 3. tRigon exemplary output for a Pearson correlation matrix. Based on multiple pathomics features for glomeruli, tubules and arteries Pearson correlation coefficients (r) were calculated. Calculations are based on the Kidney Precision Medicine Project (KPMP) cohort.

	**artery_diameters_ wall**	**glom_bowman_ sizes**	**glom_ diameters**	**glom_ distance**	**glom_ sizes**	**tuft_shape_ circularity**	**glom_tuft_ sizes**	**tubule_ diameters**	**tubule_ distance**
**artery_diameters_ wall**	1.00	0.01	0.00	0.03	0.01	0.00	0.01	0.01	-0.01
**glom_bowman_ sizes**	0.01	1	0.59	-0.01	0.71	-0.27	0.46	0.01	-0.02
**glom_ diameters**	0	0.59	1.00	-0.09	0.93	0.15	0.91	0.02	-0.03
**glom_ distance**	0.03	-0.01	-0.09	1.00	-0.06	-0.06	-0.06	0.00	-0.02
**glom_ sizes**	0.01	0.71	0.93	-0.06	1.00	-0.03	0.95	0.02	-0.03
**tuft_shape_ circularity**	0.00	-0.27	0.15	-0.06	-0.03	1.00	0.08	0.00	0.02
**glom_tuft_ sizes**	0.01	0.46	0.91	-0.06	0.95	0.08	1.00	0.02	-0.03
**tubule_ diameters**	0.01	0.01	0.02	0.00	0.02	0.00	0.02	1.00	-0.24
**tubule_ distance**	-0.01	-0.02	-0.03	-0.02	-0.03	0.02	-0.03	-0.24	1.00

Supplementary Table 4. tRigon was used to replicate an analysis from[1] reaching the same results.

**Histomics feature**	**Kruskal-Wallis test *p*-value**
NoOfLowGradeNucleiPerEpithNest	0.0002334448652544735
SizeOfEpithNuclei	1.549626562065984e-11
ComplexityOfEpithNuclBoundary	0.001118878880746352
StainingOfEpithNuclei	0.973774731872592
ChromatinClumpingOfEpithNuclei	8.557837247294508e-8

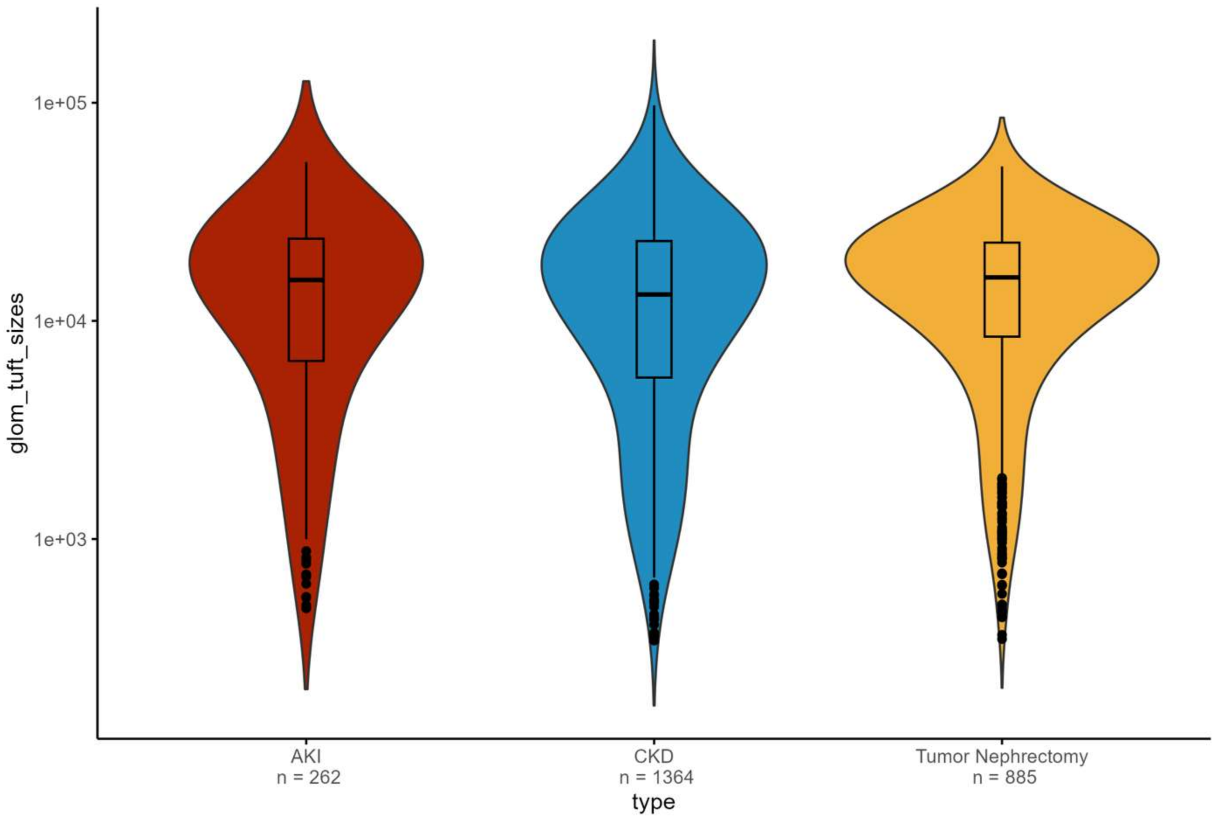

Supplementary Figure 1. tRigon exemplary output for a combined violin and boxplot. Three different sample classes from the Kidney Precision Medicine Project (KPMP) cohort were compared based on their respective glomerular tuft sizes.
AKI, acute kidney injury; CKD, chronic kidney disease; n, number of datapoints.

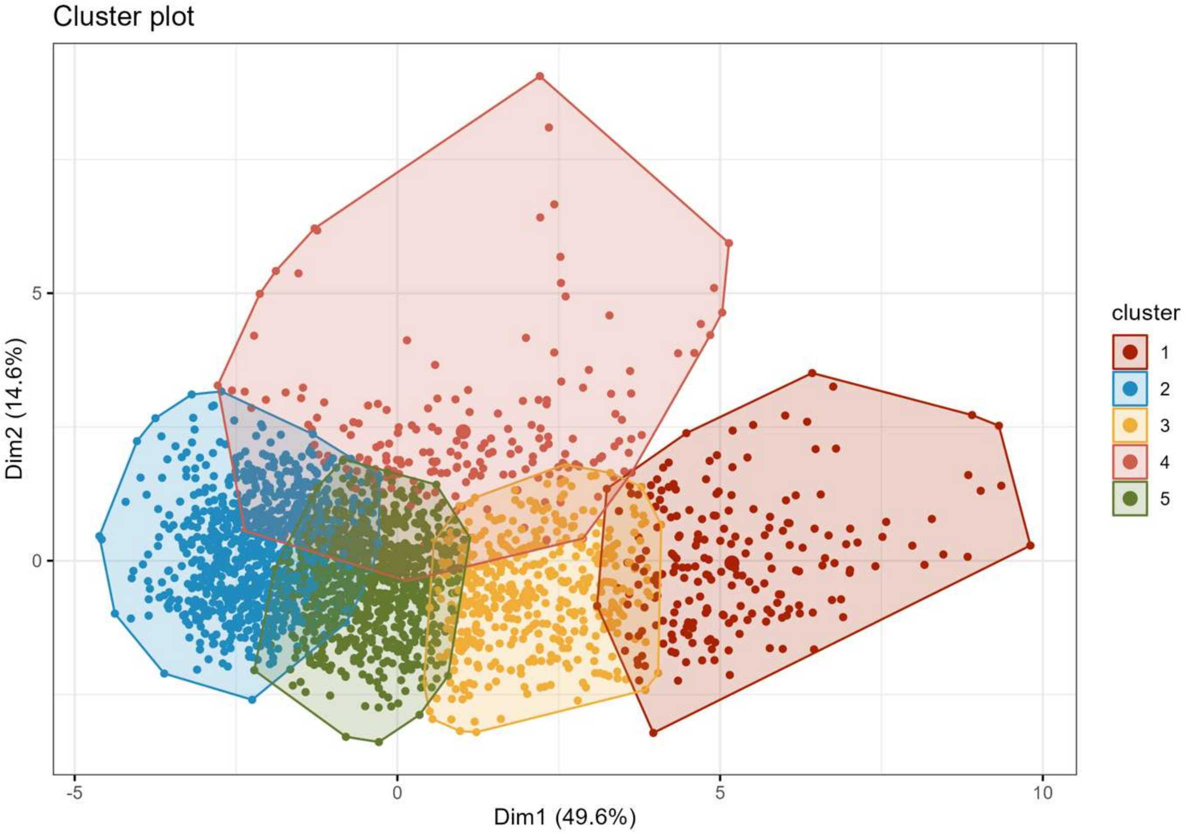

Supplementary Figure 2. tRigon exemplary output for a k-means clustering plot. 12 glomerular features of samples from the Kidney Precision Medicine Project (KPMP) cohort were clustered based on k-means clustering with 5 prespecified clusters. Additional group labels were disabled.

Dim1, dimension one; Dim2, dimension two.

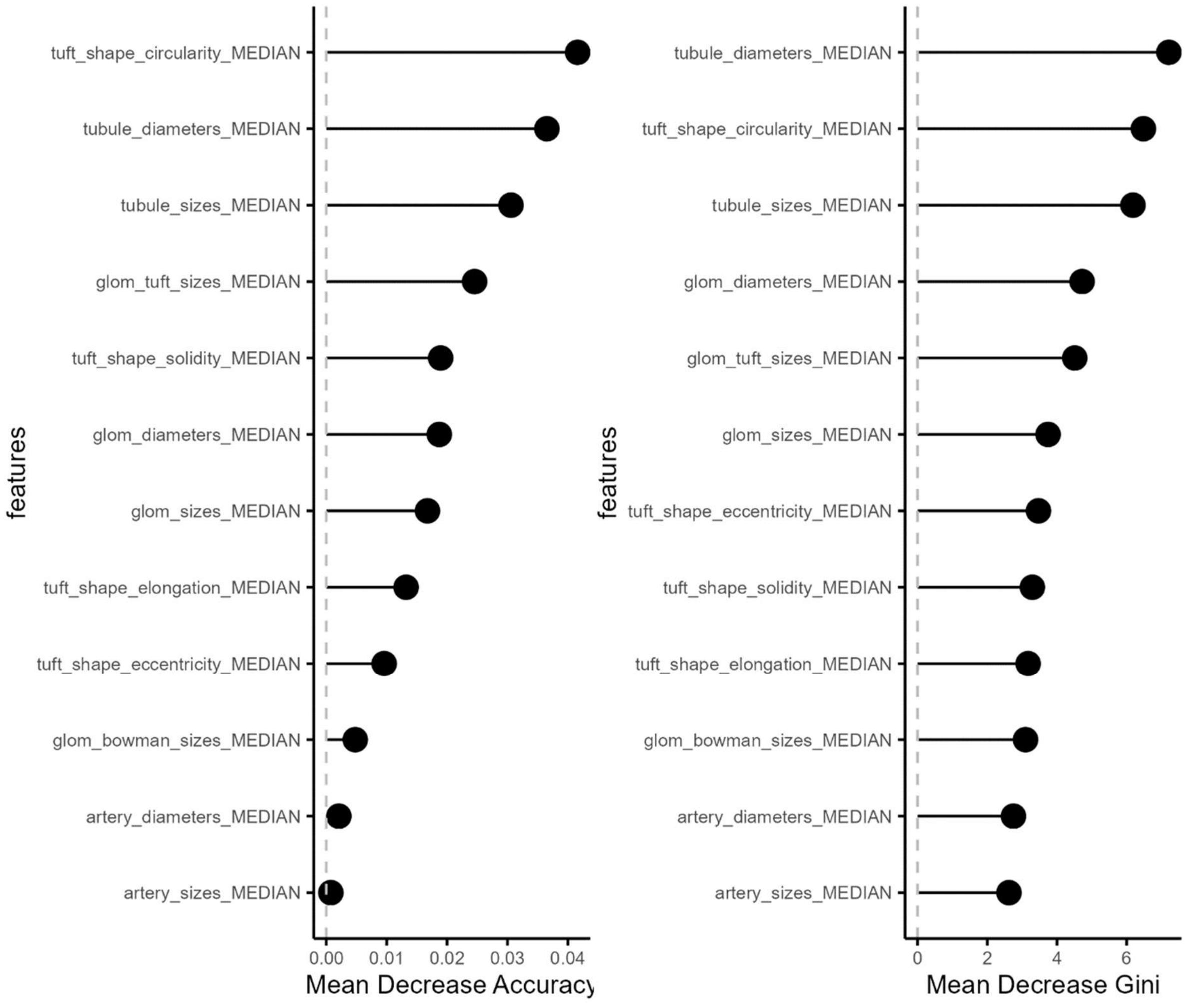

Supplementary Figure 3. tRigon exemplary output for a feature importance plot. Multiple pathomics features of glomeruli, tubules and arteries from the Kidney Precision Medicine Project (KPMP) cohort were summarized at specimen-level. Specimen medians were implemented in a recursive feature elimination based on the sample class to determine feature importance.

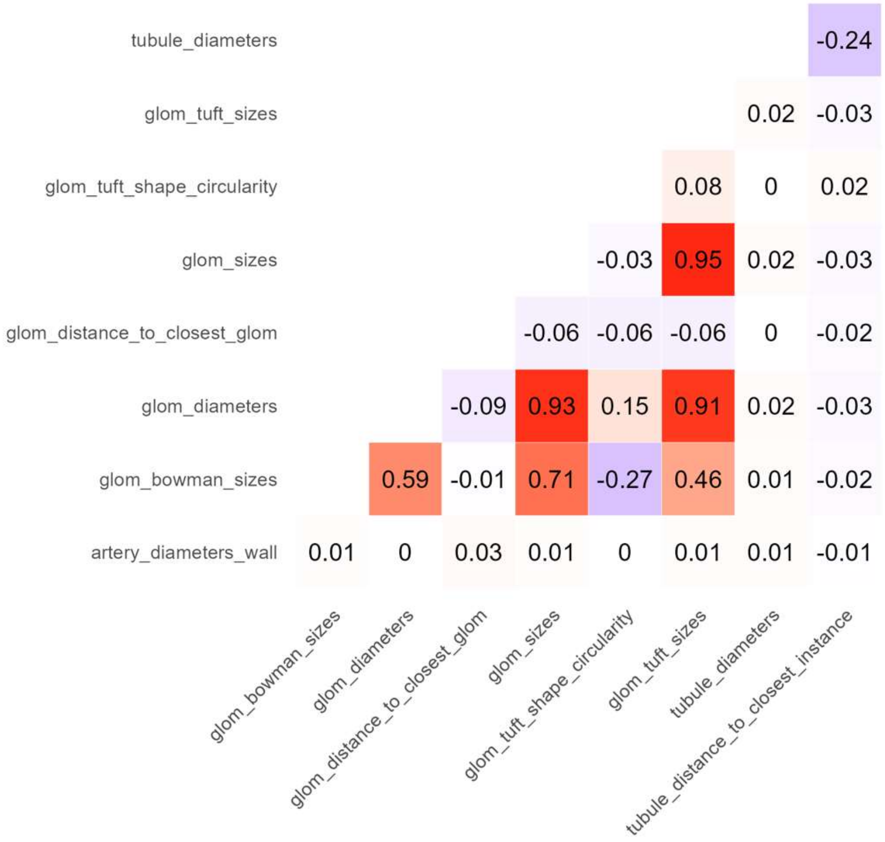

Supplementary Figure 4. tRigon exemplary output for a correlation heatmap. Pearson correlation coefficients (r) were calculated for all displayed features in the Kidney Medicine Project (KPMP) cohort.

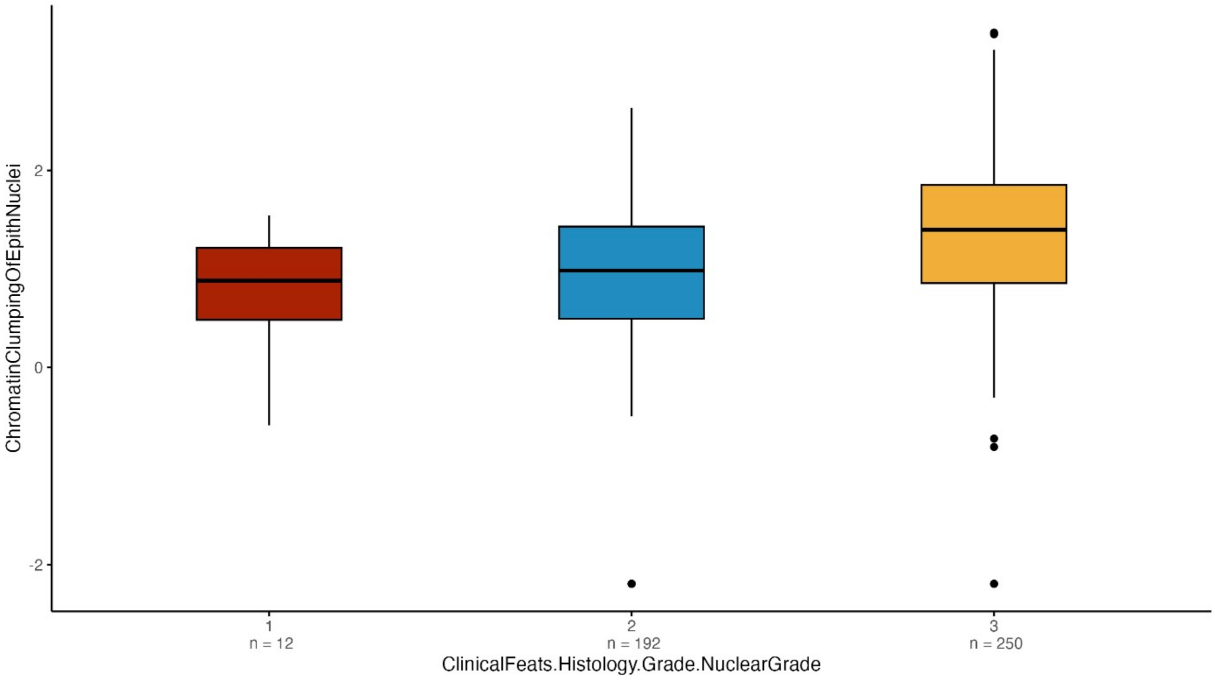

Supplementary Figure 5. tRigon based reanalysis of the feature "Chromatin Clumping of Epithelial Nuclei" stratified by tumor grade from[1].

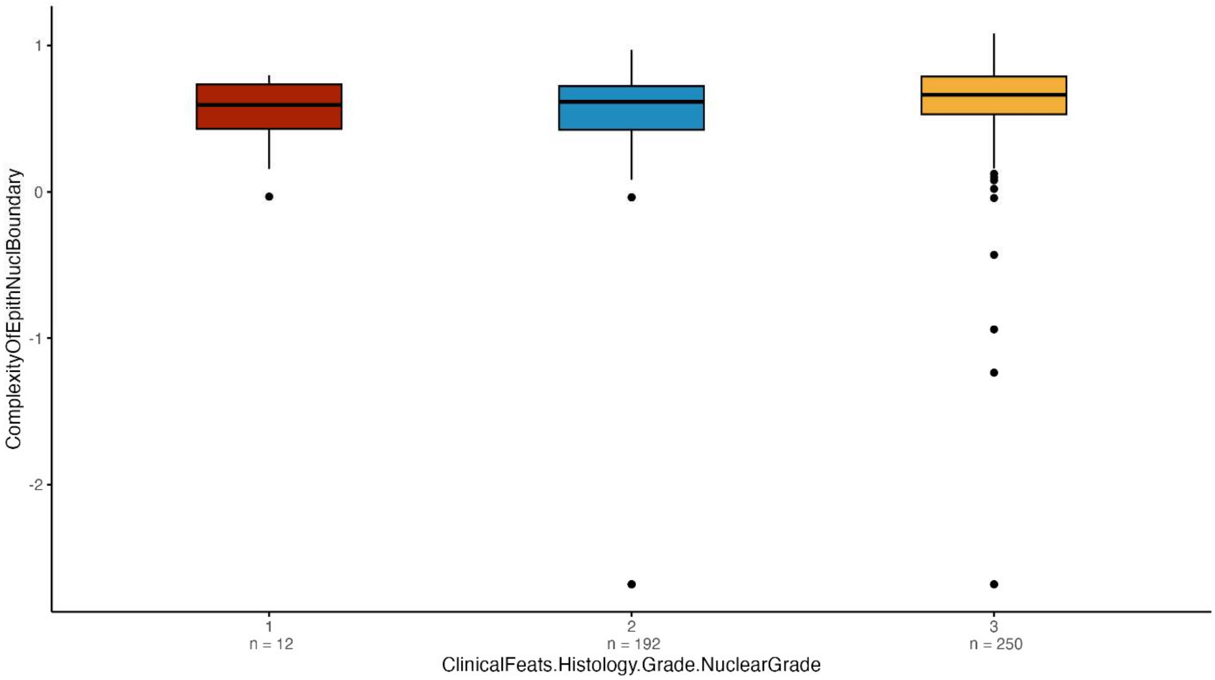

Supplementary Figure 6. tRigon based reanalysis of the feature "Complexity of Epithelial Nuclei Boundary" stratified by tumor grade from[1].

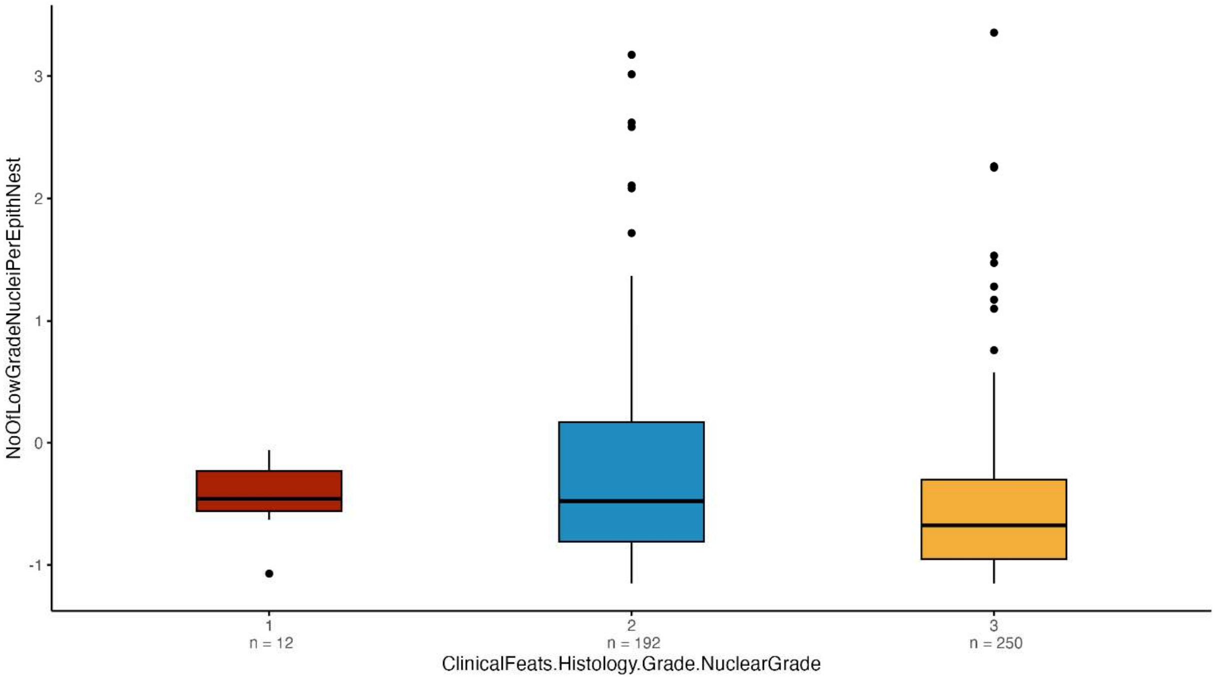

Supplementary Figure 7. tRigon based reanalysis of the feature "Number of Low Grade Nuclei Per Epithelial Nest" stratified by tumor grade from[1].

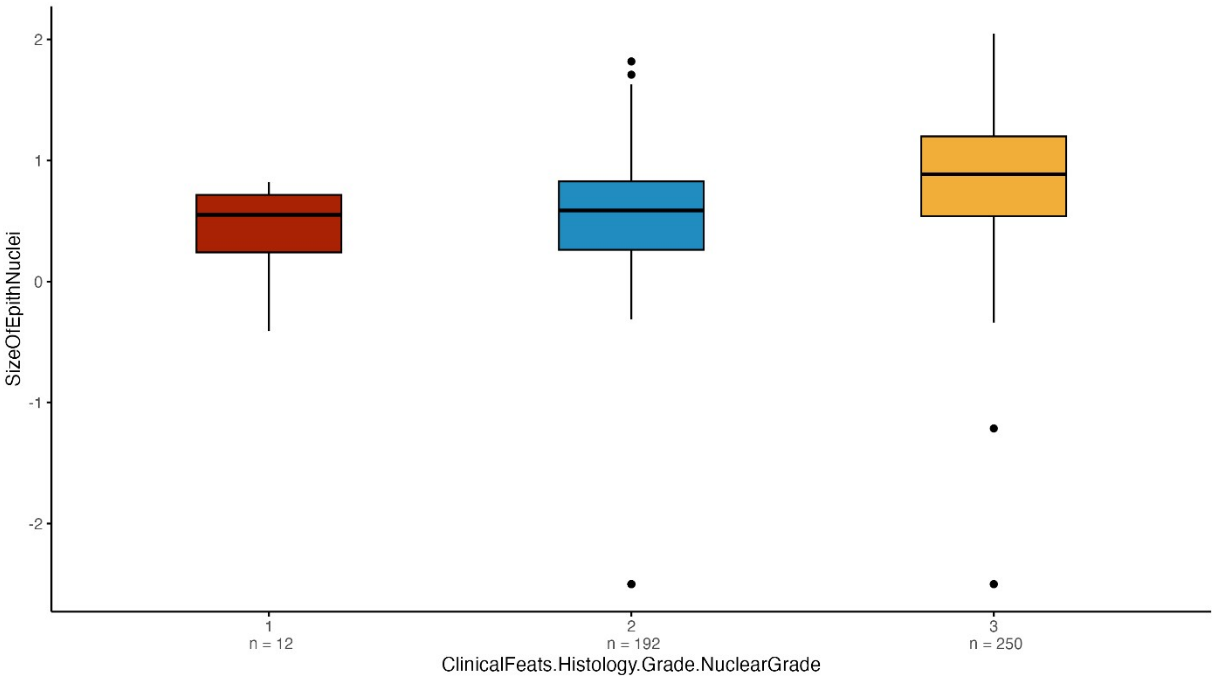

Supplementary Figure 8. tRigon based reanalysis of the feature "Size of Epithelial Nuclei" stratified by tumor grade from[1].

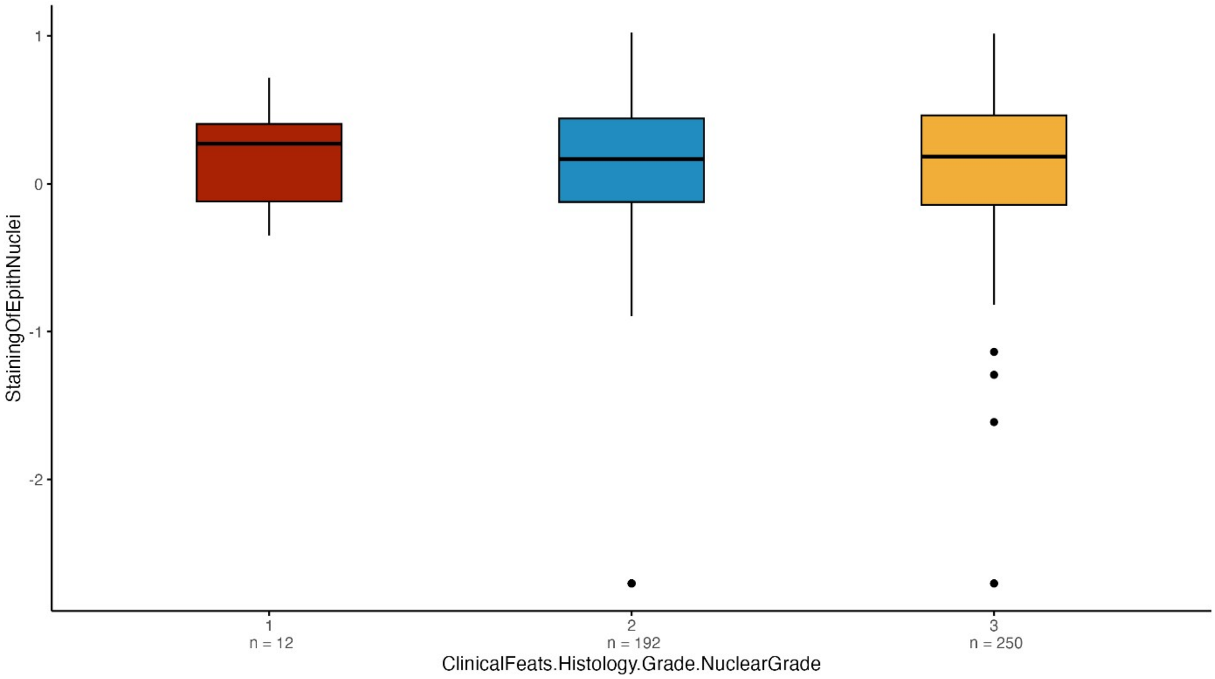

Supplementary Figure 9. tRigon based reanalysis of the feature "Staining of Epithelial Nuclei" stratified by tumor grade from[1].

Supplementary Reference:

1. Amgad, M. *et al.* A population-level digital histologic biomarker for enhanced prognosis of invasive breast cancer. *Nat. Med.* (2023) doi:10.1038/s41591-023-02643-7.
